# Supplementary material for: Simultaneous spatiotemporal super-resolution and multi-parametric fluorescence microscopy
Source: Nat Commun. 2021 Mar 19;12:1748. doi: 10.1038/s41467-021-22002-9 (PMC7979808; doi:10.1038/s41467-021-22002-9)
Supplement: Supplementary file 1 — Supplementary Information [file 41467_2021_22002_MOESM1_ESM.pdf]

Supplementary material for

Simultaneous spatiotemporal super-resolution and multi-parametric fluorescence microscopy

Jagadish Sankaran[1,4], Harikrushnan Balasubramanian[1,4], Wai Hoh Tang[2], Xue Wen Ng[1,3], Adrian Röllin[2], Thorsten Wohland[1,3]

[1]Department of Biological Sciences and NUS Centre for Bio-Imaging Sciences, National University of Singapore, Singapore, Singapore.

[2]Department of Statistics and Applied Probability, National University of Singapore, Singapore, Singapore.

[3]Department of Chemistry, National University of Singapore, Singapore, Singapore.

[4]contributed equally to the work.

***Corresponding author*:** Thorsten Wohland, twohland@nus.edu.sg

Table of Contents

1 SUPPLEMENTARY NOTE 1: INTRODUCTION 3

2 SUPPLEMENTARY NOTE 2: INSTRUMENTATION AND DATA-ANALYSIS USING THE SCMOS CAMERA 4

3 SUPPLEMENTARY NOTE 3: SUPPLEMENTARY N&B ANALYSIS 5

3.1 BRIGHTNESS CALIBRATION 5
3.2 EXPERIMENTALLY MEASURED PARAMETERS AND THEIR ERROR CALCULATIONS 7
3.2.1 Pooled mean and SEM of brightness 7
3.2.2 Errors associated with derived parameters 7
3.3 BRIGHTNESS OF EGFR 8
3.3.1 Error calculations of derived parameters 9
3.4 BRIGHTNESS OF OLIGOMERS 9
3.4.1 Error propagation of brightness of oligomers 10
3.4.2 Estimation of EGFR oligomerization 10
3.4.3 Estimation of higher oligomer species 11
3.4.4 Error propagation of equations yielding proportion of higher order oligomers 12

4 SUPPLEMENTARY NOTE 4: GPU-BASED PLUGIN 13

4.1.1 GPU Kernel 13
4.1.2 Array sizes 13
4.1.3 Comparison of processing time between CPU and GPU 13
4.1.4 Program availability 14

5 SUPPLEMENTARY NOTE 5: PSF CALIBRATION IN IMAGING FCS 15

6 SUPPLEMENTARY NOTE 6: OPTIMIZATION OF N&B ANALYSIS 17

7 SUPPLEMENTARY NOTE 7: OPTIMIZATION OF SRRF 19

8 SUPPLEMENTARY NOTE 8: FCS BASED CORRECTION STRATEGY FOR SRRF 25

8.1 USE OF FILTERED D MAP TO CORRECT THE SRRF MAP 25

9 SUPPLEMENTARY NOTE 9: ESTIMATING DIMERIZATION FRACTION OF EGFR IN LIVE CELLS USING N&B ANALYSIS 34

10 SUPPLEMENTARY NOTE 10: DIFFUSION LAW ANALYSIS OF EGFR ON LIVE CELLS 35

11 SUPPLEMENTARY NOTE 11: USE OF SCMOS IN MULTI-PARAMETRIC ANALYSIS 37

12 SUPPLEMENTARY NOTE 12: THEORETICAL ESTIMATION OF MAXIMUM BRIGHTNESS AND BRIGHTNESS RATIO OF CELLS 40

13 SUPPLEMENTARY FIGURES FOR "MODULATION OF EGFR DYNAMICS AND CLUSTERING" 41

14 APPENDIX 45

14.1 PLASMIDS UTILIZED IN THIS STUDY 45
14.2 BRIGHTNESS OF N^{TH} ORDER OLIGOMER SPECIES 46

15 SUPPLEMENTARY REFERENCES 47

1 Supplementary Note 1: Introduction

There exists a wide variety of fluorescence spectroscopy and microscopy techniques that are able to obtain either spatial super-resolution or high temporal resolution. Unfortunately, spatial super-resolution techniques often require special instrumentation that not only limits their availability but also restricts the possibility to obtain good temporal resolution.

We demonstrate a strategy to perform simultaneous multi-parametric analysis using fast and sensitive cameras (EMCCD and sCMOS) and a combination of super-resolution and spectroscopy techniques – deconvolution, super resolution optical fluctuation imaging (SOFI), super resolution radial fluctuations (SRRF) imaging to resolve actin fibrillar structures, fluorescence correlation spectroscopy (FCS) analysis to determine diffusion coefficient, number and brightness (N&B) analysis to determine particle brightness, and FCS diffusion law analysis to determine membrane organization (Fig. 1). The strategy is implemented on a total internal reflection fluorescence (TIRF) microscope. SRRF[1], a computational super resolution technique with its roots in SOFI[2] yields images resolved beyond the diffraction limit by performing a SOFI analysis on radiality stacks. Among the three computational microscopy techniques, SRRF yielded the fibres with the lowest thickness in our data sets. Further analysis was restricted only to SRRF.

Imaging fluorescence correlation spectroscopy[3,4] is a single molecule sensitive ensemble-based method that yields spatially resolved diffusion maps. A statistical analysis of fluctuations in fluorescence in each pixel of an array detector provides the autocorrelation function (ACF) in every pixel. Fitting the ACF in each pixel to theoretical models yields the diffusion coefficient (D) and the number of particles in that pixel (N). Typically, for molecules with multiple diffusing states (for instance, Lifeact in this study has a fibrillar and non-fibrillar D), a large coefficient of variation (COV; ratio of standard deviation to the mean) of D is observed. The error associated with D is reduced by selecting only the fibrillar features using TIRF image as a mask. Further refinement is performed by eliminating pixels with unusually low D not attributable to Lifeact molecules diffusing on fibres ($<0.2\ \mu m^2/s$). This diffusion map was used to eliminate artefacts from Lifeact SRRF images.

As a proof of principle, we perform a two-colour experiment with EGFR-mApple and Lifeact-EGFP in live CHO-K1 cells. Apart from fitting the ACFs to estimate the D, we also performed FCS diffusion law analysis for the determination of diffusion modes and the sub-resolution organization of the diffusing particles under investigation. For this purpose, the estimated D at various observation areas are transformed to yield the average transit time through the area. The FCS diffusion law[5,6] states that the average transit time through an observation area increases linearly with increase in observation area in the case of a freely diffusing molecule. Non-linearity in the diffusion law is typically reported by quantifying the y-intercept of an approximated linear function. A positive intercept is obtained in the case of confined diffusion.

In the case of Imaging FCS, the ACF at different lag times is determined from the time-varying intensity at each pixel. In the case of N&B[7] analysis, only the mean and variance of the time-varying fluorescence intensity function is computed. The concentration and brightness at each pixel are estimated from the computed mean and variance. To obtain proper estimations of brightness values in N&B analysis, it is important to correctly take into account the background and its variance as produced by the detector, in this case EMCCD or sCMOS cameras, as described here[8]. A comparison of the brightness of a particle with the brightness of a monomer

with the knowledge of the probability of a fluorophore to be fluorescent enables one to estimate the oligomerization state of the particle.

The results demonstrate that we can obtain super-resolution in space (~100 nm) with simultaneously high resolution in time (2 ms) from the exact same data set. This allows us to mutually correlate the various parameters and obtain new information on EGFR organization and dynamics in relation to the cytoskeleton.

2 Supplementary Note 2: Instrumentation and data-analysis using the sCMOS camera

The TIRF microscopy set up is described in the main text. Apart from the EMCCD described in the main text, here we used also an sCMOS (Sona 4.2B-11, 11 µm pixel size, 2048 × 2048 pixels, Andor, Oxford Instruments, UK) camera for measurements. The following settings were used for recording the image stacks with the sCMOS camera, the laser power was set to 100 µW for the 488 nm laser and 19 mW for the 561 nm laser. The camera settings and the laser power used for the EMCCD are provided in the main text. We recorded stacks of 50,000 frames of 150 × 300 pixels at 500 fps (for cell measurements) or 1,000 fps (for bilayer measurements). The software Andor Solis was used for image acquisition. The acquisition mode used was “kinetic series”. The pixel readout rate was 200 MHz in an overlap readout mode. The rolling electronic shuttering mode at a 12-bit dynamic range was used. The acquisition settings for the various experimental configurations are summarized in Supplementary Table 1. The point spread function (PSF) calibration measurements were performed at 500 µW laser power for the 488 nm laser and 1 mW for the 561 nm laser.

Supplementary Table 1: Acquisition parameters for the various experimental configurations

Camera	Analysis	Laser [nm]	Time per frame [ms]	No. of frames	Image pixel size [nm]	Map dimensions [Pixels]
**sCMOS**	FCS	561	2	50,000	220	71 × 71
**Supplementary**	Diffusion law	561	2	50,000	330	10 × 10
**Fig. 15**	N&B	561	20	5,000	220	71 × 71
	SRRF	488	200	500	22	710 × 710*

*The original image has a pixel size of 110 nm at 1 × 1 binning. During the SRRF analysis, virtual sub-pixels of size 22 nm are created.

In the case of ×200 magnification, we used 2 × 2 binning for Imaging FCS analysis on the EMCCD to maintain a good signal-to-noise ratio (SNR). Similarly, the sCMOS data for Imaging FCS, performed with ×100 magnification, was also binned 2 × 2. The diffusion law plot for sCMOS was performed for square binning of 3 to 7 in contrast to the EMCCD data shown in Fig. 4 where square binning of 1-5 was used. For the N&B analysis using the sCMOS camera, the pixels were 2 × 2 spatially binned and also time binned to 20 ms. Unless otherwise stated, spatial binning was not performed for SRRF microscopy, but frames were time binned up to 200 ms. All analyses were performed using the graphics processing unit (GPU) based plugin in ImageJ, described in Supplementary Note 4.

3 Supplementary Note 3: Supplementary N&B analysis

The time-varying intensity in a pixel-*I(t)* is determined by the apparent brightness (*B)* of particles and the time-varying apparent number of particles-*N(t)* in that pixel.

$$\langle I(t)\rangle = B\langle N(t)\rangle = BN \qquad \textit{Eq. 1}$$

where $\langle I(t)\rangle$ is the time-averaged intensity and N is the average number of particles in that pixel. The variance of the intensity is determined only by the variance of the number of particles within the observation volume since the brightness of fluorescent particles is assumed to be a constant within the measurement time. For any random variable X and a constant k,

$$Var(kX) = k^2 Var(X) \qquad \textit{Eq. 2}$$

Hence

$$\sigma^2 = B^2 Var\big(N(t)\big) \qquad \textit{Eq. 3}$$

where σ^2 is the variance of the fluorescence intensity in the pixel. The number of particles within the observation volume follows a Poissonian distribution. Hence the variance of the time-varying number of particles is equal to the mean of the number of particles within the observation volume.

$$\sigma^2 = B^2 N \qquad \textit{Eq. 4}$$

Substituting $\langle I(t)\rangle = BN$, we get

$$\sigma^2 = B\langle I(t)\rangle \qquad \textit{Eq. 5}$$

Rearranging the equation above, we get[8]:

$$B = \frac{\sigma^2}{\langle I(t)\rangle} = \frac{\sigma^2}{\langle I\rangle} \qquad \textit{Eq. 6}$$

$$N = \frac{\langle I(t)\rangle^2}{\sigma^2} = \frac{\langle I\rangle^2}{\sigma^2} \qquad \textit{Eq. 7}$$

Throughout the rest of the document we represent $\langle I(t)\rangle$ as $\langle I\rangle$, since the time-varying intensity is assumed to be stationary.

3.1 Brightness calibration

The oligomerization state of a protein is determined by computing the ratio of the brightness of the proteins to the monomeric state of the fluorescent protein (FP) used. Not all molecules

of FPs are fluorescent due to incomplete maturation, misfolding, photo-bleaching issues and possible dark states of the fluorescent moiety[9-11]. Hence in order to estimate the oligomerization state of a protein, one needs to estimate the proportion of FPs that are fluorescent (p). The value p is estimated by computing the brightness of two different constructs, monomeric FP and two FPs in tandem (referred to as dimeric FP). If the proportion of fluorescent FPs is p, the proportion of non-fluorescent FPs is *1-p*. Since a dimer can consist of both fluorescent and non-fluorescent molecules, there are 4 possibilities for their combination in a dimer with the following probabilities: $p^2, p(1-p), (1-p)p$ and $(1-p)^2$.

In a population consisting of N_1 monomers, the number of fluorescent molecules are pN_1. If the mean brightness of an individual fluorophore is ε, the average intensity $\langle I \rangle_m$ of the population is

$$\langle I \rangle_m = \varepsilon p N_1 \qquad \textit{Eq. 8}$$

The variance in the number of fluorescent molecules is same as the mean number of fluorescent molecules since the number of fluorescent molecules in the observation volume is Poisson distributed. The variance scales as the square of the brightness. Hence, the variance of the intensity (σ_m^2) is

$${\sigma_m}^2 = \varepsilon^2 p N_1 \qquad \textit{Eq. 9}$$

For the dimer sample with the brightness of monomer and dimer being ε and 2ε, respectively, only molecules containing at least one fluorescent FP will contribute to the intensity. Hence the average intensity of the dimer ($\langle I \rangle_d$) of N_2 molecules is written as

$$\langle I \rangle_d = 2\varepsilon p^2 N_2 + 2\varepsilon p(1-p)N_2 = 2\varepsilon p N_2 \qquad \textit{Eq. 10}$$

The variance of the intensity of the dimer (σ_d^2) is given below.

$${\sigma_d}^2 = (2\varepsilon)^2 p^2 N_2 + 2\varepsilon^2 p(1-p)N_2 = 2\varepsilon^2 p^2 N_2 + 2\varepsilon^2 p N_2 \qquad \textit{Eq. 11}$$

Substituting *Eq. 9, Eq. 10* and *Eq. 11,* in *Eq. 6*

$$B_m = \frac{{\sigma_m}^2}{\langle I \rangle_m} = \varepsilon \qquad \textit{Eq. 12}$$

$$B_d = \frac{{\sigma_d}^2}{\langle I \rangle_d} = \varepsilon(p+1) \qquad \textit{Eq. 13}$$

where B_m is the brightness of a monomer and B_d is the average brightness of dimers. Dividing *Eq. 13* by *Eq. 12* and rearranging the terms, we obtain an equation to estimate the proportion of fluorescent molecules of an FP:

$$p = \frac{B_d}{B_m} - 1 \qquad \textit{Eq. 14}$$

Denoting the ratio of the brightness of the dimer to the brightness of monomer as B_{ratio}, one obtains

$$p = B_{ratio} - 1 \qquad \textit{Eq. 15}$$

3.2 Experimentally measured parameters and their error calculations

In the case of N&B analysis, the average brightness of each cell is estimated as an average of the brightness of all pixels within the cell. The data from many such cells were pooled to obtain the population mean and error of the brightness of particular molecules in cells.

3.2.1 Pooled mean and SEM of brightness

For each cell, the arithmetic mean and standard deviation (SD) were calculated from the brightness values of pixels in an image of the cell after intensity filtering. Filtering was performed on the *B* map, to ensure that background pixels outside the cells were excluded (refer Supplementary Note 6). A typical threshold of at least 1500 counts was used. The SD was converted to the standard error of the mean (SEM).

$$SD_j = \sqrt{\frac{\sum_{i=1}^{n_j}(B_{i,j} - B_{mean_j})^2}{n_j}} \qquad Eq.\ 16$$

$$SEM_j = \frac{SD_j}{\sqrt{n_j}} \qquad Eq.\ 17$$

where $B_{i,j}$ is the brightness of an individual pixel *i* in cell *j*, B_{mean_j} is the mean brightness of the pixels in cell *j*, and n_j is the number of pixels in cell *j*. The pooled weighted arithmetic mean brightness and pooled SEM were calculated.

$$B_{pooled} = \frac{\sum_{j=1}^{N_c} n_j B_{mean_j}}{\sum_{j=1}^{N_c} n_j} \qquad Eq.\ 18$$

$$SEM_{pooled} = \sqrt{\frac{\sum_{j=1}^{N_c} SEM_j^2 + \sum_{j=1}^{N_c} \frac{\left(B_{mean_j} - B_{pooled}\right)^2}{n_j}}{N_c}} \qquad Eq.\ 19$$

where N_c is the total number of cells.

3.2.2 Errors associated with derived parameters

In the case of derived parameters, partial derivatives were used to perform error propagation of experimentally measured parameters. For the parameters defined in section 3.1,

$$B_{ratio} = \frac{B_{d,pooled}}{B_{m,pooled}} \qquad Eq.\ 20$$

$$\Delta B_{ratio} = B_{ratio}\sqrt{\left(\frac{\Delta B_{m,pooled}}{B_{m,pooled}}\right)^2 + \left(\frac{\Delta B_{d,pooled}}{B_{d,pooled}}\right)^2} \qquad Eq.\ 21$$

where, ΔB_{ratio} is the propagated SEM of the pooled dimer-to-pooled monomer brightness ratio, $B_{d,pooled}$ is the pooled mean brightness of dimer measurements, $\Delta B_{d,pooled}$ is the pooled

SEM of dimer measurements, $B_{m,pooled}$ is the pooled mean brightness of monomer measurements, and $\Delta B_{m,pooled}$ is the pooled SEM of monomer measurements.

The proportion of fluorescent molecules of an FP is obtained by subtracting a value of 1 from B_{ratio}. Hence the error associated with the proportion of FPs being fluorescent is

$$\Delta p = \Delta B_{ratio} \qquad \textit{Eq. 22}$$

where Δp is the SEM of the proportion of FPs which are fluorescent.

3.3 Brightness of EGFR

If we assume that the EGFR sample has a mixture of both monomers and dimers the overall average intensity ($\langle I \rangle_E$) is the sum of the contributions from the monomers and the dimers (*Eqs. 8* and *10*).

$$\langle I \rangle_E = \varepsilon p N_1 + 2\varepsilon p N_2 \qquad \textit{Eq. 23}$$

The overall variance of the intensity of EGFR (${\sigma_E}^2$) is the sum of the variances of the intensities of the monomer and dimer components (*Eqs. 10* and *12*). Hence,

$${\sigma_E}^2 = \varepsilon^2 p N_1 + 2\varepsilon^2 p^2 N_2 + 2\varepsilon^2 p N_2 \qquad \textit{Eq. 24}$$

Substituting *Eq. 24* in *Eq. 6*, and simplifying the equation,

$$B_E = \frac{\varepsilon(N_1 + 2pN_2 + 2N_2)}{N_1 + 2N_2} \qquad \textit{Eq. 25}$$

where B_E is the brightness of EGFR. Dividing *Eq. 25* by *Eq. 12*

$$\frac{B_E}{B_m} = r = \frac{N_1 + 2pN_2 + 2N_2}{N_1 + 2N_2} \qquad \textit{Eq. 26}$$

where r is the ratio of the brightness of EGFR to the brightness of a monomer. If the monomer EGFR population fraction is f and dimer EGFR population fraction is 1-f,

$$f = \frac{N_1}{N_1 + N_2} \qquad \textit{Eq. 27}$$

$$f = \frac{2(p - r + 1)}{2p - r + 1} \qquad \textit{Eq. 28}$$

$$1 - f = e_d = \frac{r - 1}{2p - r + 1} \qquad \textit{Eq. 29}$$

where e_d is the EGFR dimer fraction. Rewriting the EGFR dimer fraction in terms of EGFR molecules present as dimers,

$$m_e = \frac{2e_d}{e_d + 1} = \frac{r - 1}{p} \qquad \textit{Eq. 30}$$

where m_e is the mean fraction of EGFR molecules present as dimers.

3.3.1 Error calculations of derived parameters

Using partial derivatives, the errors associated with the derived parameters in section 3.3 were determined.

$$\Delta r = r\sqrt{\left(\frac{\Delta B_{m,pooled}}{B_{m,pooled}}\right)^2 + \left(\frac{\Delta B_{E,pooled}}{B_{E,pooled}}\right)^2} \qquad \textit{Eq. 31}$$

where Δr is the propagated SEM of the EGFR-to-monomer brightness ratio, $B_{E,pooled}$ is the pooled mean brightness of EGFR measurements, and $\Delta B_{E,pooled}$ is the pooled SEM of EGFR measurements.

$$\Delta e_d = \frac{2\sqrt{p^2\Delta r^2 + (r-1)^2\Delta p^2}}{(2p - r + 1)^2} \qquad \textit{Eq. 32}$$

where Δe_d is the SEM of the EGFR dimer fraction.

$$\Delta m_e = \frac{2\Delta e_d}{(e_d + 1)^2} = \frac{1}{p}\sqrt{\left(\frac{r-1}{p}\right)^2 \Delta p^2 + \Delta r^2} \qquad \textit{Eq. 33}$$

where Δm_e is the SEM of the mean fraction of EGFR molecules present as dimers.

3.4 Brightness of oligomers

In the case where the sample contains oligomers of order 'n'

$$\langle I \rangle_n = N_n \sum_{i=1}^{n} (i\varepsilon)\binom{n}{i} p^i (1-p)^{n-i} \qquad \textit{Eq. 34}$$

$$\sigma_n{}^2 = N_n \sum_{i=1}^{n} (i\varepsilon)^2 \binom{n}{i} p^i (1-p)^{n-i} \qquad \textit{Eq. 35}$$

where $\sigma_n{}^2$ is the variance of the intensity of the oligomer, $\langle I \rangle_n$ is the average intensity of oligomer sample, N_n is the number of oligomer molecules. Substituting the variance and intensity in *Eq. 6*

$$B_n = \frac{N_n \sum_{i=1}^{n} (i\varepsilon)^2 \binom{n}{i} p^i (1-p)^{n-i}}{N_n \sum_{i=1}^{n} i\varepsilon \binom{n}{i} p^i (1-p)^{n-i}} = \frac{N_n \varepsilon^2 \sum_{i=1}^{n} i^2 \binom{n}{i} p^i (1-p)^{n-i}}{N_n \varepsilon \sum_{i=1}^{n} i \binom{n}{i} p^i (1-p)^{n-i}} = \frac{N_n \varepsilon^2 np\big((n-1)p + 1\big)}{N_n \varepsilon np} \qquad \textit{Eq. 36}$$

A detailed derivation is provided in the appendix (section 14.2). Thus,

$$B_n = \varepsilon[(n-1)p+1] \quad \textit{Eq. 37}$$

where B_n is the brightness of oligomers[10] of order n. Division by *Eq. 12* results in

$$r_{n/m} = \frac{B_n}{B_m} = (n-1)p+1 \quad \textit{Eq. 38}$$

3.4.1 Error propagation of brightness of oligomers

Using error propagation, the errors associated with the brightness of oligomer and the ratio of oligomer brightness to monomer brightness were determined.

$$\Delta B_n = \sqrt{\left((n-1)p+1\right)^2 \Delta B_m^2 + B_m^2 (n-1)^2 \Delta p^2} \quad \textit{Eq. 39}$$

$$\Delta r_{n/m} = (n-1)\Delta p \quad \textit{Eq. 40}$$

3.4.2 Estimation of EGFR oligomerization

For an EGFR sample containing a mixture of oligomers up to an oligomer of order n, we obtain

$$B_{E,oligo} = \frac{\sum_{i=1}^{n} N_i \sum_{j=1}^{i} (j\varepsilon)^2 \binom{i}{j} p^j (1-p)^{i-j}}{\sum_{i=1}^{n} N_i \sum_{j=1}^{i} j\varepsilon \binom{i}{j} p^j (1-p)^{i-j}} \quad \textit{Eq. 41}$$

where $B_{E,oligo}$ is the brightness of the EGFR in oligomeric state, N_i is the number of oligomer molecules of order-i.

Assuming a mix of EGFR monomers, dimers and trimers only, where N_1, N_2 and N_3 are the number of monomers, dimers and trimers, respectively,

$$B_{E,trimer} = \frac{\varepsilon(N_1 + 2N_2 + 2pN_2 + 3N_3 + 6pN_3)}{N_1 + 2N_2 + 3N_3} \quad \textit{Eq. 42}$$

where $B_{E,trimer}$ is the brightness of EGFR in trimeric state.

The ratio of the brightness of EGFR trimer and that of the monomer is

$$r_{E,trimer} = \frac{B_{E,trimer}}{B_{monomer}} = \frac{N_1 + 2N_2 + 2pN_2 + 3N_3 + 6pN_3}{N_1 + 2N_2 + 3N_3} \quad \textit{Eq. 43}$$

where $r_{E,trimer}$ is the ratio of the brightness of the EGFR trimer to the brightness of the monomer.

Let m, d and t be the fraction of monomers, dimers and trimers, respectively, then

$$m = 1 - d - t = \frac{N_1}{N_1 + N_2 + N_3} \quad \textit{Eq. 44}$$

$$d = \frac{N_2}{N_1 + N_2 + N_3} \quad \text{Eq. 45}$$

$$t = \frac{N_3}{N_1 + N_2 + N_3} \quad \text{Eq. 46}$$

$$r_{E,trimer} = \frac{1 + d + 2pd + 2t + 6pt}{1 + d + 2t} \quad \text{Eq. 47}$$

Rewriting $[m; d; t]$ in terms of $r_{E,trimer}$

$$\left[1 - d - t; d; \frac{1 + d + 2pd - r_{E,trimer}(1 + d)}{2r_{E,trimer} - 2 - 6p}\right] \quad \text{Eq. 48}$$

In absence of dimers:

$$\left[1 - t; 0; \frac{r_{E,trimer} - 1}{6p - 2r_{E,trimer} + 2}\right] \quad \text{Eq. 49}$$

In absence of trimers:

$$\left[1 - d; \frac{r_{E,trimer} - 1}{2p - r_{E,trimer} + 1}; 0\right] \quad \text{Eq. 50}$$

It is important to note that a complete solution can be obtained only for two oligomer species. Hence the solutions shown above indicate special cases where either the trimer or the dimer is absent. If there are more than two kinds of oligomers, complete solutions are possible only when more measurement parameters are available. Therefore, we provide only apparent oligomer percentages with the understanding that they represent a minimal model of monomers and one oligomer species that are consistent with the experimental data.

3.4.3 Estimation of higher oligomer species

When higher order oligomers are present, a complete solution cannot be obtained. There are two equations and hence we solve only for a special case consisting of monomers and the minimum order oligomer required for the observed brightness. As derived in section 3.4, the ratio of brightness of an oligomer to that of a monomer is quantified as $(n - 1)p + 1$. For ratios in the range of $[1, p + 1]$, the population consists of at least dimers. For ratios in the range of $[p + 1, 2p + 1]$, the population consists of at least trimers. Depending on the ratio, the minimum order oligomer is determined. Denoting N_1 as the number of monomers, N_n as the number of minimum order oligomers, $f_{oligomer}$ as the fraction of minimum order of oligomers

As shown in section 3.4,
Contribution of monomer to the mean - $N_1 \varepsilon p$
Contribution of minimum order oligomer to the mean - $N_n \varepsilon n p$
Contribution of monomer to the variance - $N_1 \varepsilon^2 p$
Contribution of minimum order oligomer to the variance - $N_n \varepsilon^2 n p\big((n - 1)p + 1\big)$

$$B_{E,min-oligomer+monomer} = \frac{\varepsilon\left(N_1 + N_n n\big((n-1)p+1\big)\right)}{N_1 + nN_n} \qquad \text{Eq. 51}$$

$$r_{E,mom} = \frac{B_{E,min-oligomer+monomer}}{B_m} = \frac{N_1 + N_n n\big((n-1)p+1\big)}{N_1 + nN_n} \qquad \text{Eq. 52}$$

$$f_{oligomer} = \frac{N_n}{N_1 + N_n} \qquad \text{Eq. 53}$$

$$r_{E,mom} = \frac{1 + f_{oligomer}\big(n\big((n-1)p+1\big)-1\big)}{1 + f_{oligomer}(n-1)} \qquad \text{Eq. 54}$$

$$f_{oligomer} = \frac{r_{E,mom} - 1}{(n-1)\big(np + 1 - r_{E,mom}\big)} \qquad \text{Eq. 55}$$

Assuming there are a total of E molecules of EGFR, the number of monomers present as oligomer and monomers are $nEf_{oligomer}$ and $E\big(1 - f_{oligomer}\big)$. Hence the fraction of EGFR molecules present as oligomers is

$$e_{oligomer} = \frac{nf_{oligomer}}{f_{oligomer}(n-1) + 1} = \frac{r_{E,mom} - 1}{p(n-1)} \qquad \text{Eq. 56}$$

3.4.4 Error propagation of equations yielding proportion of higher order oligomers

Error propagation was performed on equations derived in section 3.4.3.

$$\Delta f_{oligomer} = \frac{n}{(n-1)(np - r_{E,mom} + 1)^2}\sqrt{p^2\Delta r_{E,mom}^2 + (r_{E,mom} - 1)^2\Delta p^2} \qquad \text{Eq. 57}$$

$$\Delta e_{oligomer} = \frac{n\Delta f_{oligomer}}{\big(f_{oligomer}(n-1) + 1\big)^2} = \frac{1}{p(n-1)}\sqrt{\left(\frac{r-1}{p}\right)^2 \Delta p^2 + \Delta r^2} \qquad \text{Eq. 58}$$

Throughout the manuscript and rest of this document, unless specified otherwise, the terms “dimer fraction” and “oligomer fraction” will refer to the fraction of EGFR molecules present as dimers (m_e) and oligomers ($e_{oligomer}$), respectively.

4 Supplementary Note 4: GPU-based plugin

The input for the Imaging FCS plugin in ImageJ is a 16-bit tiff image stack. ACFs are calculated at every pixel, and fitted with an appropriate model. The GPU code parallelizes the calculation of the correlation functions and their fits.

The described GPU acceleration has been performed for the computation of ACFs, N&B analysis and computation of FCS diffusion laws. In the case of the computation of ACFs, the binning, bleach correction, and calculation of the correlations are all performed on the GPU. The fitting is performed in the GPU using the open source GPUfit program[12]. The flow chart showing the sequence of calculations is shown in Supplementary Fig. 1a.

In order to run calculations on an NVIDIA GPU installed in a machine with a 64-bit Windows or Linux operating system, *agpufit.dll* or *libagpufit.so* is loaded in Windows or Linux respectively. Upon running Imaging FCS, availability of a CUDA runtime environment is checked using *isCudaAvailable()*.

If the criteria are not met, the program will perform calculations on the central processing unit (CPU) instead. The input to the Imaging FCS plugin is a stack of images of dimensions [*r*, *c*, *t*] where *r*, *c* and *t* are the number of rows, columns and timepoints respectively. The bridge from Imaging FCS to CUDA code, which is written in C++, is made possible through the Java Native Interface (JNI). The JNI static functions are coded in *gpufitImFCS.java*.

4.1.1 GPU Kernel

Depending upon the user parameters, the entire or a part of the image stack of dimensions [*win*, *hin*, t] is transferred to the GPU. ACF calculation on the GPU is parallelized along each pixel of the image. The parameters *wtemp* and *htemp* (refer Supplementary Fig. 1 for definitions) determines CUDA kernel size, i.e. the number of blocks per grid, which is denoted with prefix *gridSize* in the CUDA code. The number of threads per block, which is denoted with prefix *blockSize*, is set at 16 × 16 × 1. Due to the limited resources of the GPU and the size of the data we cannot maximise the *blockSize* at 32 × 32 × 1. On the same token, parameter *power_of_two_n_points* in *Info::configure()* function is increased by a factor of 4 in comparison to the original *gpufit* code because of the complexity of the *ACF_1D CUDA* kernel fitting function.

4.1.2 Array sizes

The JNI API *SetFloatArrayRegion* has limited output array sizes. We therefore limited 2 × 2 binning of stacks 50,000 frames to 96 pixels × 96 pixels. Binning of larger stacks, especially full frames of 128 pixels × 128 pixels × 50,000 frames, are binned by the CPU. This is not an intrinsic limit of the method, but a technical issue of the GPU memory size. If an error is encountered while doing a calculation in GPU mode, the program will then perform the calculation on the CPU.

4.1.3 Comparison of processing time between CPU and GPU

Random walk simulations as described here[2] were used to simulate a data set which was used for estimating the processing time using a CPU and GPU (Supplementary Fig. 1).

4.1.4 Program availability

The Imaging FCS 1.52. ImageJ plugin is included in the ImageJ update site, and is also available at https://github.com/ImagingFCS/Imaging_FCS_1_52-,
or at http://www.dbs.nus.edu.sg/lab/BFL/imfcs_image_j_plugin.html.

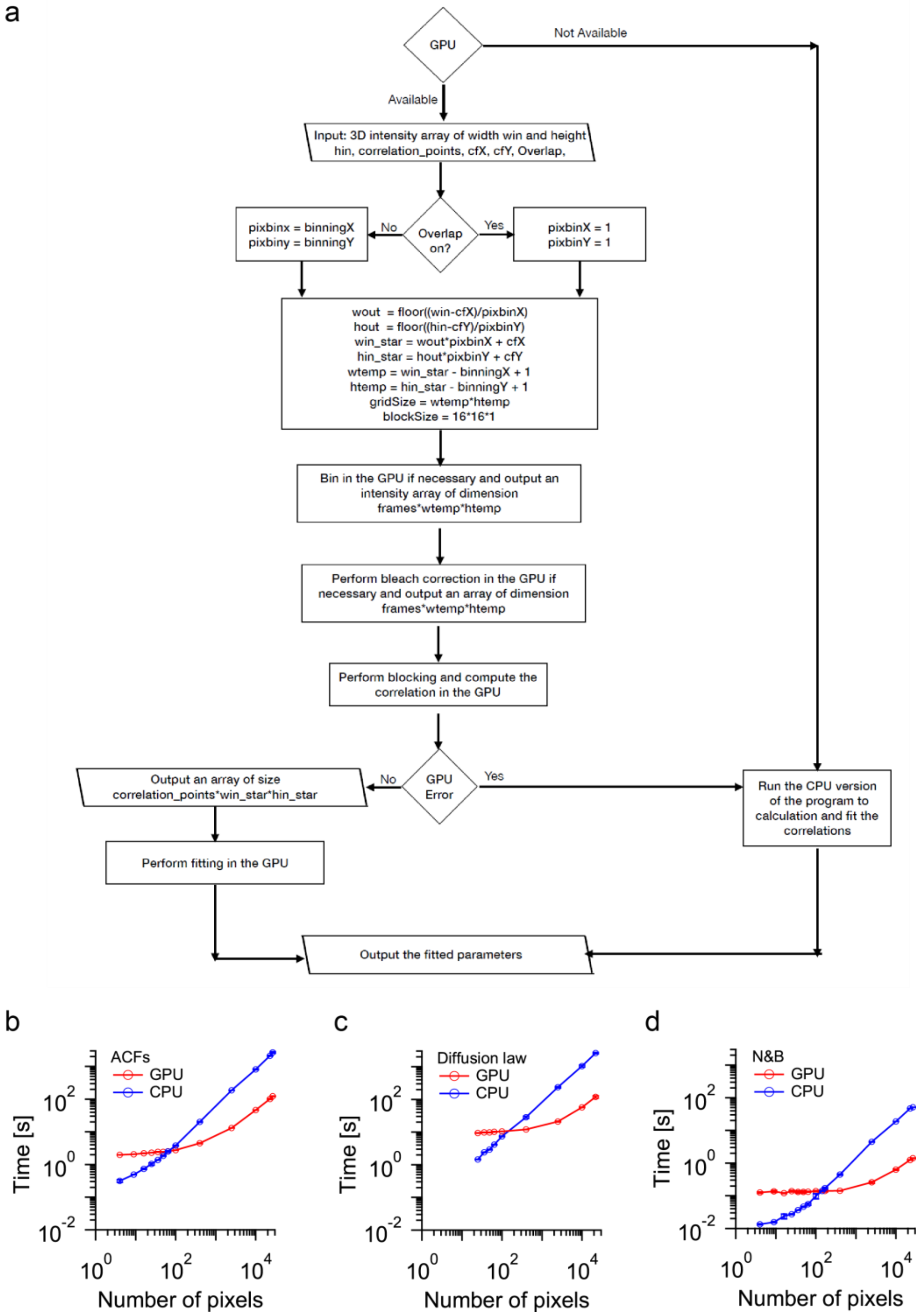

Supplementary Fig. 1: GPU based processing: **(a)** Flow chart describing the flow of control on the GPU, and the computation time comparison between GPU and CPU for **(b)** ACFs fitting, **(c)** FCS diffusion law and **(d)** N&B.

5 Supplementary Note 5: PSF calibration in Imaging FCS

The instrumental parameters in the fitting model in Imaging FCS are the pixel size and the PSF. The PSF is determined in Imaging FCS based on the fact that the estimated *D* is independent of the observation area used, as determined by pixel binning, since *D* is an intrinsic molecular property.

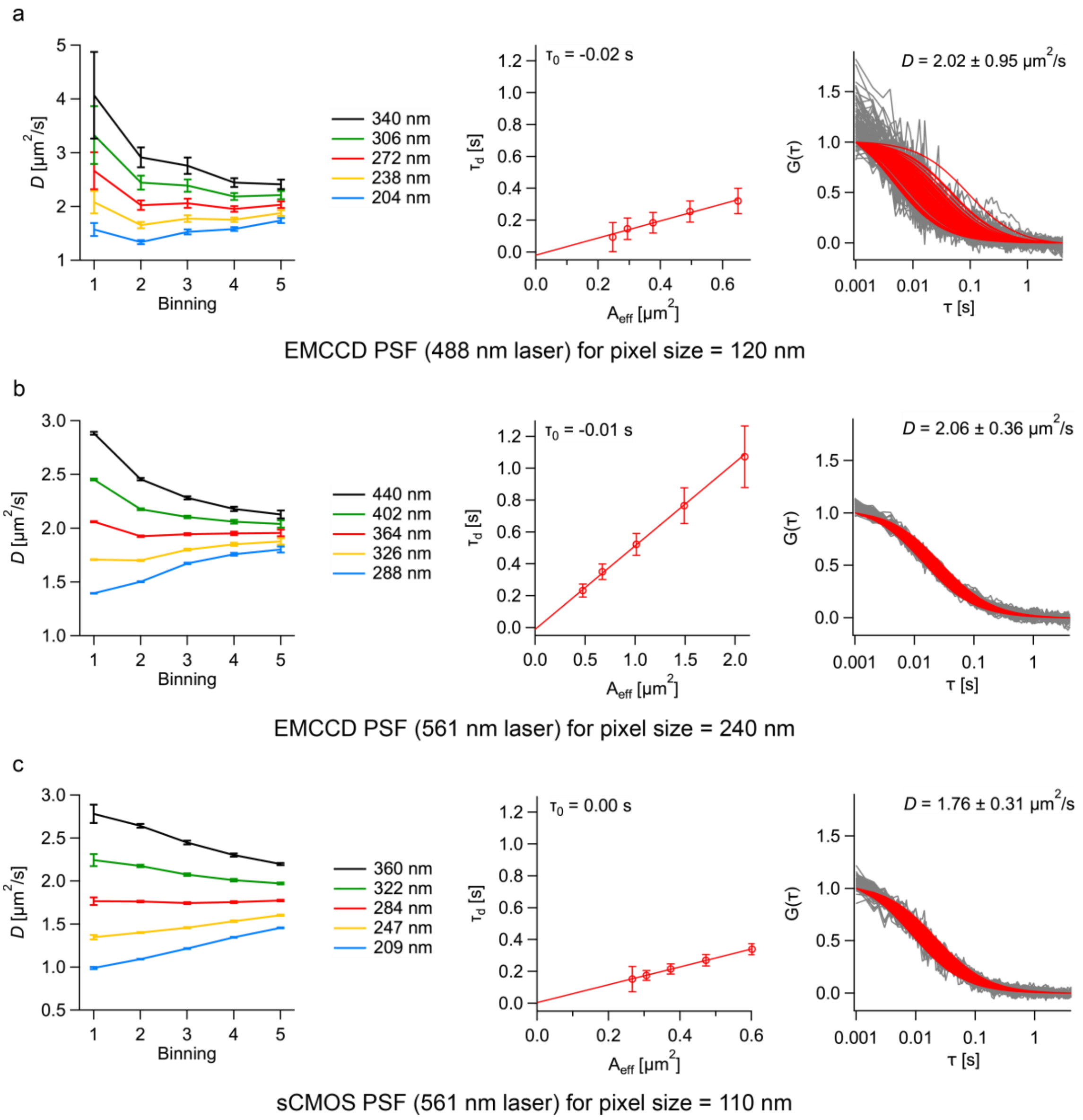

Supplementary Fig. 2: PSF calibration and diffusion law plots: Representative PSF calibration, diffusion law plots and normalized ACFs for **(a)** the EMCCD camera at ×200 magnification (120 nm pixel size in object space) at a wavelength of 488 nm, **(b)** the EMCCD camera at ×100 magnification (240 nm pixel size in object space) at a wavelength of 561 nm, and **(c)** the sCMOS camera at ×100 magnification (110 nm pixel size in object space) at a wavelength of 561 nm are shown. The detailed experimental configurations are provided in Supplementary Table 2. The procedure to obtain the PSF calibration plot is described in the manuscript. Briefly, the *D* at various bin sizes (n = 400 pixels at 1 × 1 binning) are determined for different values of the PSF. The PSF which yields a *D* independent of the bin area is the PSF of the system. The normalized ACFs are shown at 1 × 1 binning for (b) (pixel size = 240 nm; n = 400 pixels) and at 2 × 2 binning for (a) and (c) (pixel size = 240 nm and 220 nm, respectively; n = 100 pixels). All values are reported as Mean ± SD.

The D at various bin sizes are determined for different values of the PSF. The PSF which yields a D independent of the bin area is the PSF of the system. For the purpose of the calibration we define the PSF as

$$PSF = \frac{\omega_{xy}\lambda}{NA} \qquad \textit{Eq.59}$$

where ω_{xy} is the dimensionless scaling factor, λ is the wavelength of emission, and NA is the numerical aperture of the detection objective.

All three setups used in this study yield similar D. However, measurements using the 488 nm laser have a larger error than those performed using the 561 nm laser. This is attributed to the lower SNR of the ACFs obtained from the lipilight488 dye excited by the 488 nm laser (Supplementary Fig. 2).

Supplementary Table 2: *1/e²* radii of various experimental configurations

	**Laser [nm]**	**Camera**	**P_{chip} [µm]**	**Obj**	**Additional magnification**	**P_{mag} [nm]**	**PSF [nm]**	**D_{avg} [µm²/s]**
**(a)**	**488**	EMCCD	24	100×	×2	120	272	1.91 ± 0.90
**(b)**	**561**	EMCCD	24	100×	None	240	364	2.06 ± 0.39
**(c)**	**561**	sCMOS	11	100×	None	110	284	1.77 ± 0.37

P_{chip}-Pixel size on chip; **P_{mag}**-Pixel size after magnification; **Obj**-Objective. The D_{avg} reported is at 1 × 1 binning for **(b)** (**P_{mag}** = 240 nm) and at 2 × 2 binning for **(a)** and **(c)** (**P_{mag}** = 240 nm and 220 nm, respectively) from 5 different measurements.

6 Supplementary Note 6: Optimization of N&B analysis

A threshold is chosen to separate the cellular and non-cellular regions in the image. The effect of varying the threshold is shown in Supplementary Fig. 3d-h. Insufficient thresholding retains some of the cellular exterior while excessive thresholding leads to reduction of cellular areas. Note that thresholding also leads to an increase in average brightness as low brightness regions are excluded. Once thresholding becomes excessive cell areas are also excluded but with little change in brightness. This is expected as excessive thresholding only removes regions of lower concentration on the cell but brightness is independent of concentration.

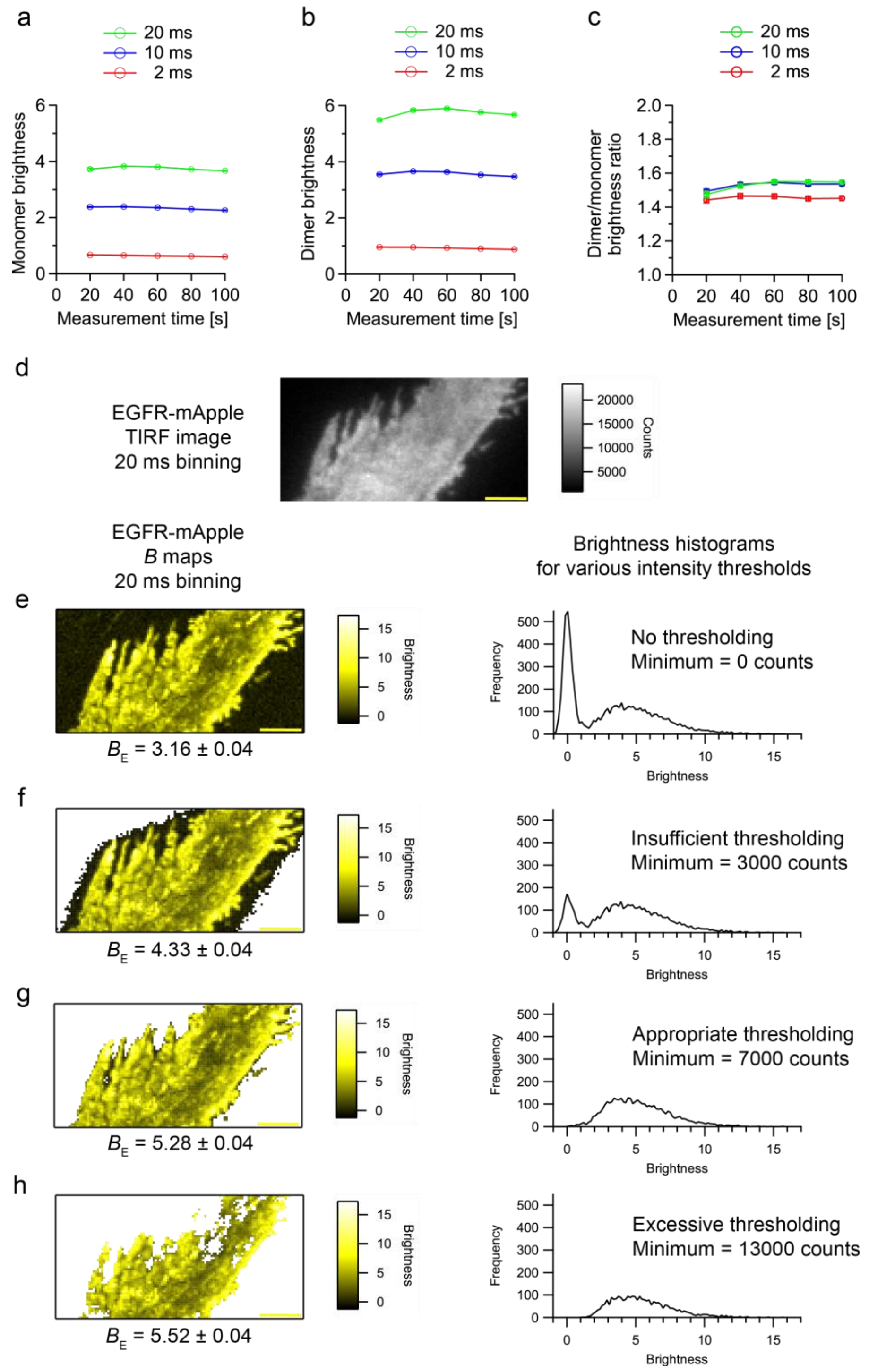

Supplementary Fig. 3: Number and Brightness analysis – Effects of exposure time, measurement time and intensity thresholding on brightness. (a) and (b) The mean brightness of PMT-mApple and PMT-mApple$_2$ is plotted against the total measurement time, for different exposure times (2 ms, 10 ms, 20 ms). **(c)** The brightness ratio of PMT-mApple$_2$/PMT-mApple is plotted against total measurement time, for different exposure times (2 ms, 10 ms, 20 ms). Each point is an average of 3 different cell measurements for both PMT-mApple and PMT-mApple$_2$. The number of pixels in each cell at each acquisition time is different due to differential intensity filtering in each case, but each cell had at least 1850 and at most 6250 valid pixels. (**d**) TIRF image of a CHO-K1 cell labelled with EGFR-mApple after 20 ms time binning at ×100 magnification. In panels (e)-(h), example *B* maps after different intensity thresholding are shown on the left. The corresponding histogram of the *B* values are shown on the right. Thresholding is done on all cells in this study to differentiate the cell from the background. **(e)** B_E map and histogram without thresholding. All pixels, including the background, are retained on the B_E map. The histogram shows two peaks with the background pixels as a prominent peak on the left. The average B_E value is underestimated. **(f)** B_E map and histogram after insufficient thresholding of ≥3000 counts. Some pixels from the background are still retained on the B_E map and appear as a small peak on the left in the histogram. The average B_E value is underestimated. **(g)** B_E map and histogram after appropriate thresholding of ≥7000 counts. Almost all pixels from the background are eliminated on the B_E map and a single peak is seen in the histogram. The average B_E value is representative of the cell alone. **(h)** B_E map and histogram after excessive thresholding of ≥13000 counts. Many pixels from the cell are eliminated on the B_E map. Mean ± SEM is reported here. The scale bars shown in yellow measure 5 µm in images (d)-(h).

7 Supplementary Note 7: Optimization of SRRF

SRRF was optimized using the actin structure in the green wavelength channel. Data show that the size of structural details in SRRF improves with time binning and decreasing pixel size in sample space. At 2 ms time resolution and a pixel size of 120 nm, the full width at half maximum (FWHM) recovered by SRRF for actin structures is 259 ± 42 nm. Upon time binning to 200 ms, this improves by 3.6-fold to 72 ± 7 nm (Supplementary Fig. 4). In the case of a pixel size of 240 nm, time binning to 200 ms led to a 1.7-fold improvement in the FWHM from 260 ± 30 nm to 157 ± 26 nm (Supplementary Fig. 5). Further binning in time does not improve the FWHM but is prone to the creation of artefacts. The resolution in SRRF images has been measured using Fourier ring correlation (FRC)[13,14] and peak to peak distance (P2P). In our study, the FRC and P2P were found to be 90 nm and 136 nm (Supplementary Table 3), respectively, for actin fibres measured using a pixel size of 120 nm at 200 ms time binning. A merge of TIRF, SRRF at 2 and 200 ms depicts the decrease in FWHM of the actin fibre (Supplementary Fig. 4e). We also observed that increase in pixel size from 120 nm to 240 nm led to an increase in the estimate of FWHM of actin fibres (Supplementary Figs. 4d and 4f).

A choice of parameters which leads to a decrease in FWHM of bright fibres leads to missing SRRF fibres of low TIRF intensity. Parameters which retain most of the TIRF features in SRRF lead to an increase in FWHM of bright fibres in SRRF. Thus there is a trade-off in how SRRF parameters are selected, as is correct for all computational super-resolution or deconvolution algorithms. The advantage in our application is that the users can choose parameters that lead to images that can be corroborated by the imaging of dynamics.

We also observered that there is some patterned noise visible in the images. The patterned noise is more prominent in the deconvolution image (Supplementary Fig. 6). We attribute this patterned noise to the fact that we have used the raw data from the cameras without performing any correction. If necessary, patterned noise could be removed by applying correction as described here[15].

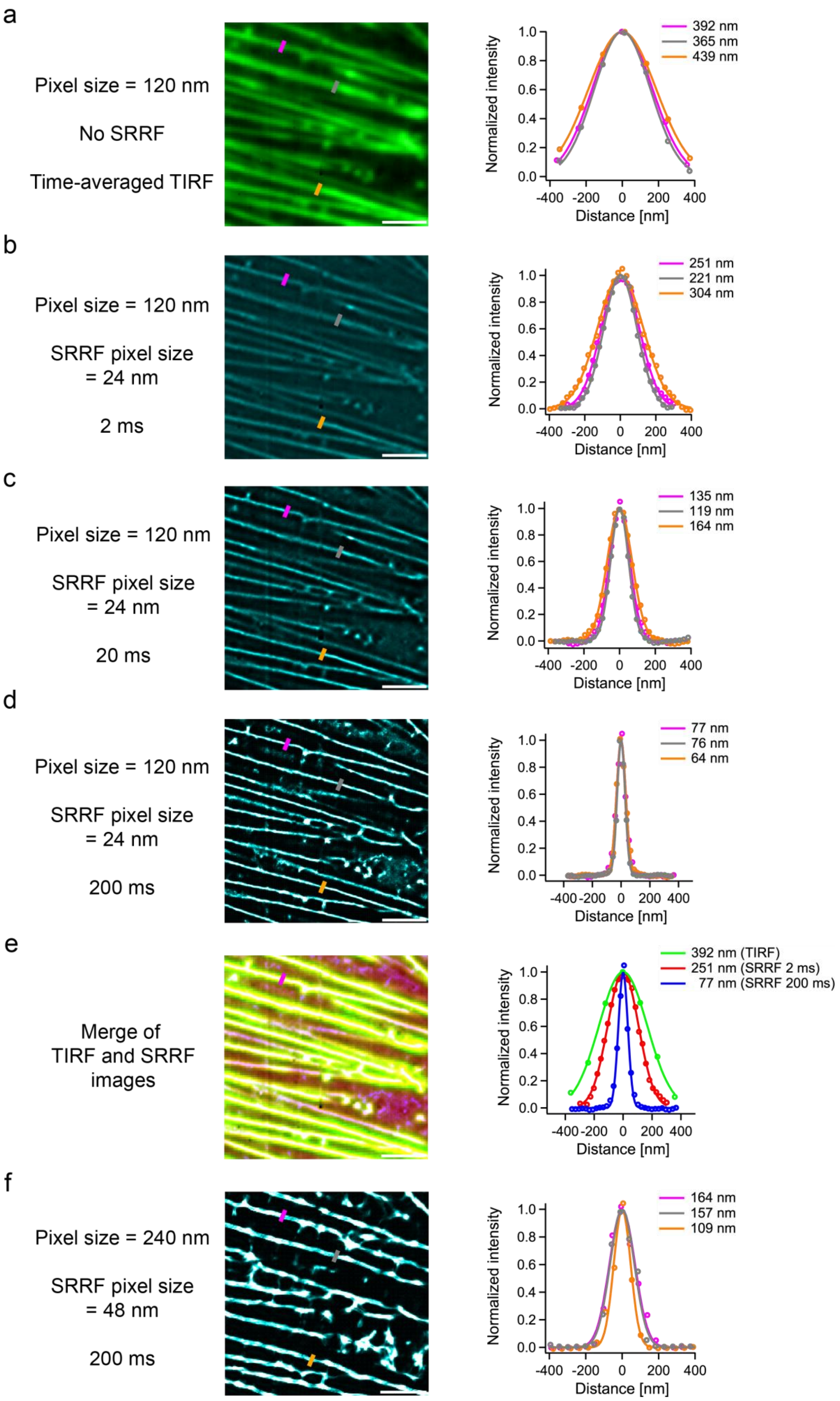
a
Pixel size = 120 nm
No SRRF
Time-averaged TIRF
392 nm
365 nm
439 nm
Normalized intensity
Distance [nm]
b
Pixel size = 120 nm
SRRF pixel size
= 24 nm
2 ms
251 nm
221 nm
304 nm
Normalized intensity
Distance [nm]
c
Pixel size = 120 nm
SRRF pixel size
= 24 nm
20 ms
135 nm
119 nm
164 nm
Normalized intensity
Distance [nm]
d
Pixel size = 120 nm
SRRF pixel size
= 24 nm
200 ms
77 nm
76 nm
64 nm
Normalized intensity
Distance [nm]
e
Merge of
TIRF and SRRF
images
392 nm (TIRF)
251 nm (SRRF 2 ms)
77 nm (SRRF 200 ms)
Normalized intensity
Distance [nm]
f
Pixel size = 240 nm
SRRF pixel size
= 48 nm
200 ms
164 nm
157 nm
109 nm
Normalized intensity
Distance [nm]

Supplementary Fig. 4: Time binning and magnification improves spatial resolution in SRRF: In panels (a)-(e), images from TIRF or SRRF as indicated are shown on the left. Normalized intensity profiles across actin fibres at three different positions (indicated by magenta, grey, and orange lines on the cell image) are shown on the right. All reported values in the figure are the FWHM of Gaussian fits to the intensity profiles: **(a**) TIRF image of a CHO-K1 cell labelled with Lifeact-EGFP at ×200 magnification. **(b)** SRRF image of the 2 ms data shown in image (a). **(c)** and **(d)** show the SRRF images after time binning of the 2 ms data shown in image (a) to 20 ms and 200 ms, respectively. **(e)** Merged TIRF and SRRF images shown in a, b and d in green, red and blue, respectively. The white pixels in this figure are common to all three images. The yellow pixels are those common only to TIRF and SRRF at 2 ms. The TIRF pixels not present in any of the SRRF images are shown in green. The histogram on the right is plotted for the magenta line in the various maps. **(f)** SRRF image and histogram generated after time binning of the 2 ms data of the same area at ×100 magnification to 200 ms. The FWHM (mean ± SD) of Gaussian Fits to the normalized intensity profiles from each image (*n* = 3 positions on the fibres) is as follows: (a) 399 ± 37 nm, (b) 259 ± 42 nm, (c) 139 ± 23 nm, (d) 72 ± 7 nm, and (f) 143 ± 30 nm. The pixel sizes reported are after magnification (refer Table 3). The scale bars shown in white measure 2.5 µm in images (a)-(f).

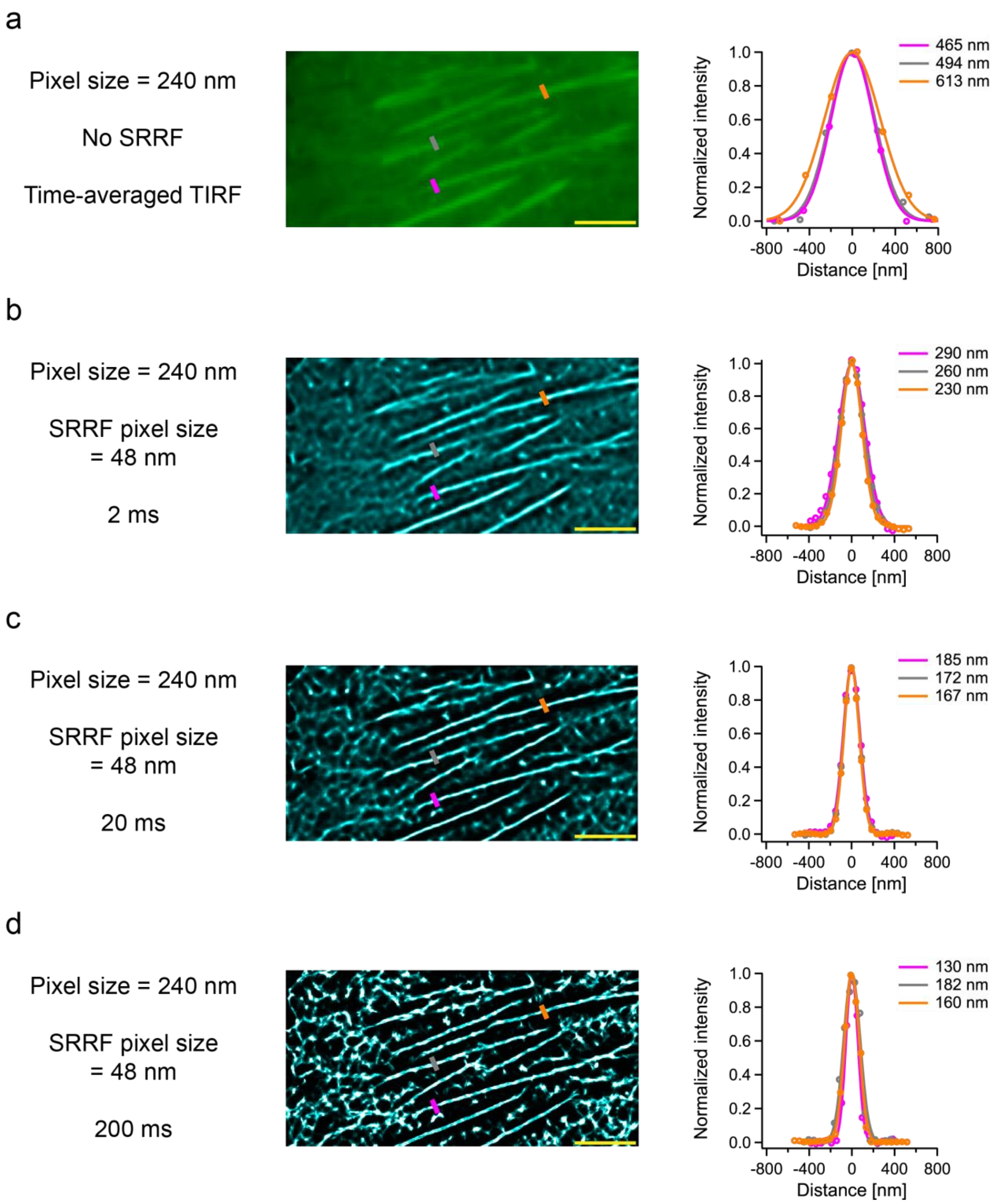

Supplementary Fig. 5: Time binning improves spatial resolution in SRRF at pixel size of 240 nm: In panels (a)-(d), images from TIRF or SRRF as indicated are shown on the left. Normalized intensity profiles across actin fibres at three different positions (indicated by magenta, grey, and orange lines on the cell image) are shown on the right. All reported values in the figure are the FWHM of Gaussian fits to the intensity profiles. **(a)** TIRF image of a CHO-K1 cell labelled with Lifeact-EGFP at ×100 magnification (same cell shown in Fig. 4). **(b)** SRRF image of the 2 ms data shown in image (a). **(c)** and **(d)** show the SRRF images after time binning of the 2 ms data shown in image (a) to 20 ms and 200 ms, respectively. The FWHM (mean ± SD) of Gaussian Fits to the normalized intensity profiles from each image (n = 3 positions on the fibres) is as follows: (a) 524 ± 79 nm, (b) 260 ± 30 nm, (c) 175 ± 9 nm, and (d) 157 ± 26 nm. The pixel sizes reported are after magnification (refer Table 3). The scale bars shown in yellow measure 5 µm in images (a)-(d).

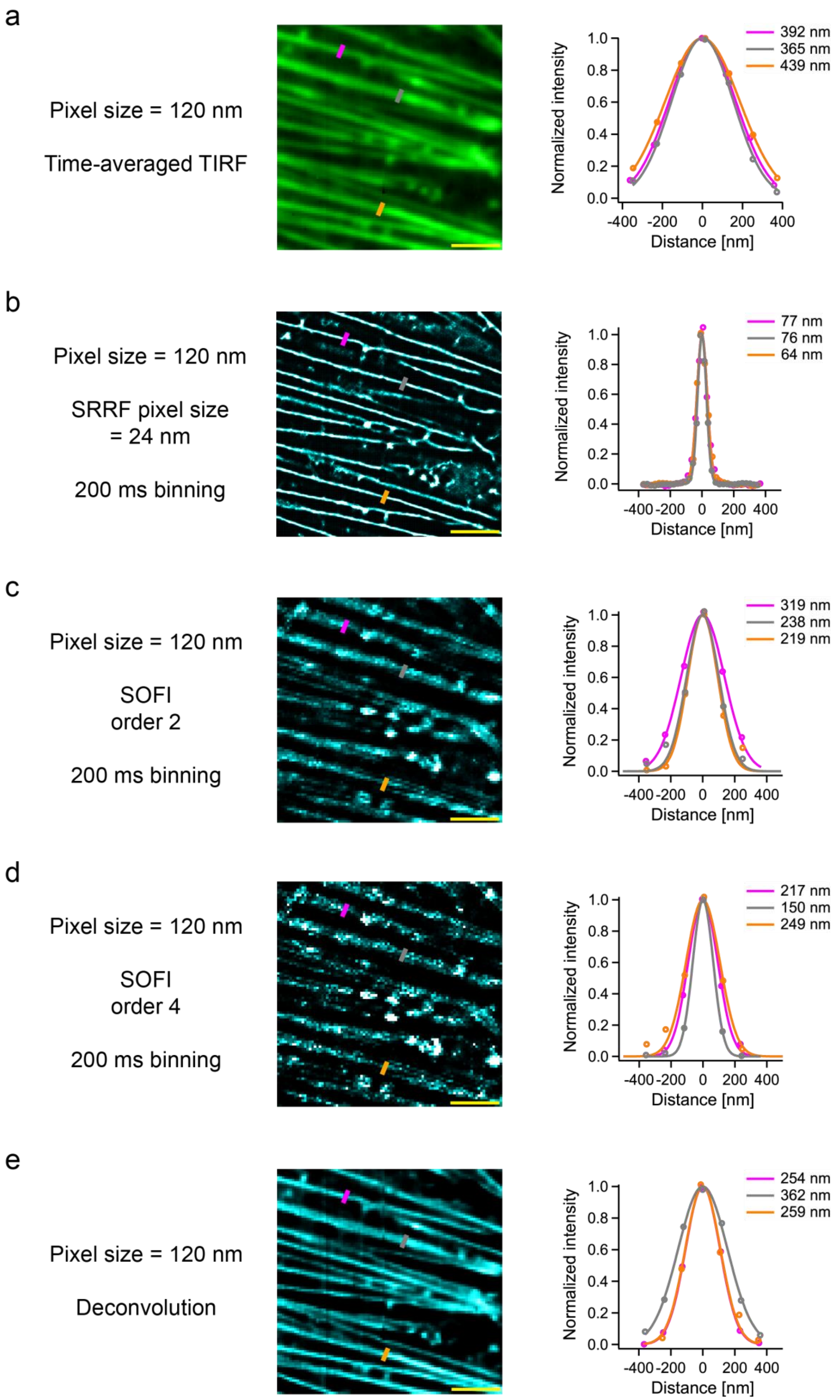
a
Pixel size = 120 nm
Time-averaged TIRF
Normalized intensity
Distance [nm]
392 nm
365 nm
439 nm
b
Pixel size = 120 nm
SRRF pixel size = 24 nm
200 ms binning
Normalized intensity
Distance [nm]
77 nm
76 nm
64 nm
c
Pixel size = 120 nm
SOFI order 2
200 ms binning
Normalized intensity
Distance [nm]
319 nm
238 nm
219 nm
d
Pixel size = 120 nm
SOFI order 4
200 ms binning
Normalized intensity
Distance [nm]
217 nm
150 nm
249 nm
e
Pixel size = 120 nm
Deconvolution
Normalized intensity
Distance [nm]
254 nm
362 nm
259 nm

Supplementary Fig. 6: Comparison of SRRF with SOFI and deconvolution: In panels (a)-(e), images from TIRF, SRRF, SOFI or deconvolution as indicated are shown on the left. Normalized intensity profiles across actin fibres at three different positions (indicated by magenta, grey, and orange lines on the cell image) are shown on the right. All reported values in the figure are the FWHM of Gaussian fits to the intensity profiles **(a**) TIRF image of a CHO-K1 cell labelled with Lifeact-EGFP at ×200 magnification. **(b)** SRRF image of the 2 ms data shown in image (a). **(c)** and **(d)** show the SOFI images of order 2 and 4 respectively. **(e)** Image obtained after the application of deconvolution algorithm. The FWHM (mean ± SD) of Gaussian Fits to the normalized intensity profiles from each image (n = 3 positions on the fibres) is as follows: (a) 399 ± 37 nm, (b) 72 ± 7 nm, (c) 259 ± 43 nm, (d) 205 ± 41 nm, and (e) 292 ± 50 nm. The pixel sizes reported are after magnification (refer Table 3). The scale bars shown in yellow measure 2.5 µm in images (a)-(e).

Supplementary Table 3: FRC and P2P measures of resolution

Camera	Pixel size in SRRF [nm]	Mean FRC [nm]	P2P [nm]	Area on SRRF image	Based on figure
**EMCCD**	48 nm	136 ± 24	240 ± 48	200 × 200	Supplementary Fig. 4f*
**EMCCD**	24 nm	90 ± 24	136 ± 50	460 × 460	Supplementary Fig. 4d

*P2P map not shown

8 Supplementary Note 8: FCS based correction strategy for SRRF

In this paper, we simultaneously perform FCS and SRRF and hence we utilize parameters derived from FCS to correct for SRRF artefacts. Firstly, we observed that the COV of the diffusion map was 113% ($D_{\mathrm{L}(2)}$ = 0.48 ± 0.54 μm^2/s, Supplementary Fig. 7). Application of a TIRF mask led to an improvement in the precision of estimate of *D* ($D^{\mathrm{TIRF}}_{\mathrm{L},on(2)}$ = 0.58 ± 0.48 μm^2/s, COV-83%). Intensity filtering by the TIRF image removed off-fibre regions. We thresholded the *D* to remove the effects of bright and slowly diffusing Lifeact aggregates. After application of *D*-based thresholding, the final estimate of *D* was found to be $D^{\mathrm{TIRF},D}_{\mathrm{L,on}(2)}$ = 0.77 ± 0.43 μm^2/s (COV-56%). The evolution of *D* at various stages of filtering is shown for four different cells (Supplementary Table 4).

Supplementary Table 4: Diffusion coefficient and number of particles after each filtering step

Cell	D	Mean ± SD μm^2/s	% COV	Number of particles	Mean ± SD per pixel	% COV	Pixels	
1	$D_{\mathrm{L}(2)}$	0.48 ± 0.54	113	$N_{\mathrm{L}(2)}$	1309 ± 1043	80	$n_{\mathrm{L}(2)}$	2115
	$D^{\mathrm{TIRF}}_{\mathrm{L,on}(2)}$	0.58 ± 0.48	83	$N^{\mathrm{TIRF}}_{\mathrm{L,on}(2)}$	970 ± 521	54	$n^{\mathrm{TIRF}}_{\mathrm{L,on}(2)}$	1503
	$D^{\mathrm{TIRF},D}_{\mathrm{L,on}(2)}$	0.77 ± 0.43	56	$N^{\mathrm{TIRF,D}}_{\mathrm{L,on}(2)}$	804 ± 344	43	$n^{\mathrm{TIRF,D}}_{\mathrm{L,on}(2)}$	1099
2	$D_{\mathrm{L}(2)}$	0.48 ± 0.70	146	$N_{\mathrm{L}(2)}$	1919 ± 1519	79	$n_{\mathrm{L}(2)}$	1517
	$D^{\mathrm{TIRF}}_{\mathrm{L,on}(2)}$	0.62 ± 0.45	73	$N^{\mathrm{TIRF}}_{\mathrm{L,on}(2)}$	1306 ± 528	40	$n^{\mathrm{TIRF}}_{\mathrm{L,on}(2)}$	1151
	$D^{\mathrm{TIRF},D}_{\mathrm{L,on}(2)}$	0.67 ± 0.43	64	$N^{\mathrm{TIRF,D}}_{\mathrm{L,on}(2)}$	1261 ± 481	38	$n^{\mathrm{TIRF,D}}_{\mathrm{L,on}(2)}$	1063
3	$D_{\mathrm{L}(2)}$	0.17 ± 0.26	153	$N_{\mathrm{L}(2)}$	2146 ± 1195	56	$n_{\mathrm{L}(2)}$	2116
	$D^{\mathrm{TIRF}}_{\mathrm{L,on}(2)}$	0.25 ± 0.30	120	$N^{\mathrm{TIRF}}_{\mathrm{L,on}(2)}$	2005 ± 866	43	$n^{\mathrm{TIRF}}_{\mathrm{L,on}(2)}$	1332
	$D^{\mathrm{TIRF},D}_{\mathrm{L,on}(2)}$	0.46 ± 0.31	67	$N^{\mathrm{TIRF,D}}_{\mathrm{L,on}(2)}$	1994 ± 662	33	$n^{\mathrm{TIRF,D}}_{\mathrm{L,on}(2)}$	678
4	$D_{\mathrm{L}(2)}$	0.24 ± 0.32	133	$N_{\mathrm{L}(2)}$	1515 ± 679	45	$n_{\mathrm{L}(2)}$	1599
	$D^{\mathrm{TIRF}}_{\mathrm{L,on}(2)}$	0.26 ± 0.32	123	$N^{\mathrm{TIRF}}_{\mathrm{L,on}(2)}$	1436 ± 589	41	$n^{\mathrm{TIRF}}_{\mathrm{L,on}(2)}$	1396
	$D^{\mathrm{TIRF},D}_{\mathrm{L,on}(2)}$	0.47 ± 0.34	72	$N^{\mathrm{TIRF,D}}_{\mathrm{L,on}(2)}$	1313 ± 414	32	$n^{\mathrm{TIRF,D}}_{\mathrm{L,on}(2)}$	771

8.1 Use of filtered D map to correct the SRRF map

We investigate SRRF artefacts and categorized them into two types – clusters and off-fibre artefacts. A small amount of aggregation occurs in most protein overexpression systems. These aggregates of freely diffusing, off-fibre localized Lifeact contribute to artefacts in SRRF. We see that the artefact area appears as a region of higher intensity in the brightness map (Supplementary Fig. 8b). The presence of aggregates is also corroborated by FCS analysis. The intensity trace is characterized by two broad spikes at 30 and 65 s. Such a behaviour is exhibited by aggregates diffusing through the observation volume. Upon computing and fitting the ACFs, the diffusion coefficient is $D^{\mathrm{TIRF,D}}_{\mathrm{L,off}(2)}$= 0.04 μm^2/s, indicating that those are aggregates. As a control measurement, the intensity trace and the ACF are shown for a pixel located on a fibre (Supplementary Fig. 8f). Unlike the cluster region, the intensity trace does not contain any spikes.

The SNR of ACFs corresponding to pixels located on off-fibre artefacts was low (Supplementary Fig. 8g and h). The use of TIRF mask removed such artefacts since the intensity of such pixels were lower than the chosen threshold. The importance of TIRF mask is highlighted by the fact that the artefact in Supplementary Fig. 8h was removed only by the use of TIRF mask but not by *D* thresholding. Our strategy allows for the removal of both off-fibre artefacts and clusters. The stepwise evolution of the SRRF image is shown in Supplementary Figs. 8c-e.

In the case of dual-colour measurements and sCMOS measurements we used only TIRF as a mask to correct SRRF for the green channel. The ACFs in the green channel have lower SNR due to some loss of signal in dual-colour configuration due to extra optical elements and the restriction of the wavelength range of detection, and thus were not used for filtering. Although this is not as effective as the strategy with *D* thresholding included, it removes off-fibre artefacts (Fig. 4f) and still allows the investigation of the correlation between the ACFs of the red channel (EGFR) with structure in the green channel (actin cytoskeleton).

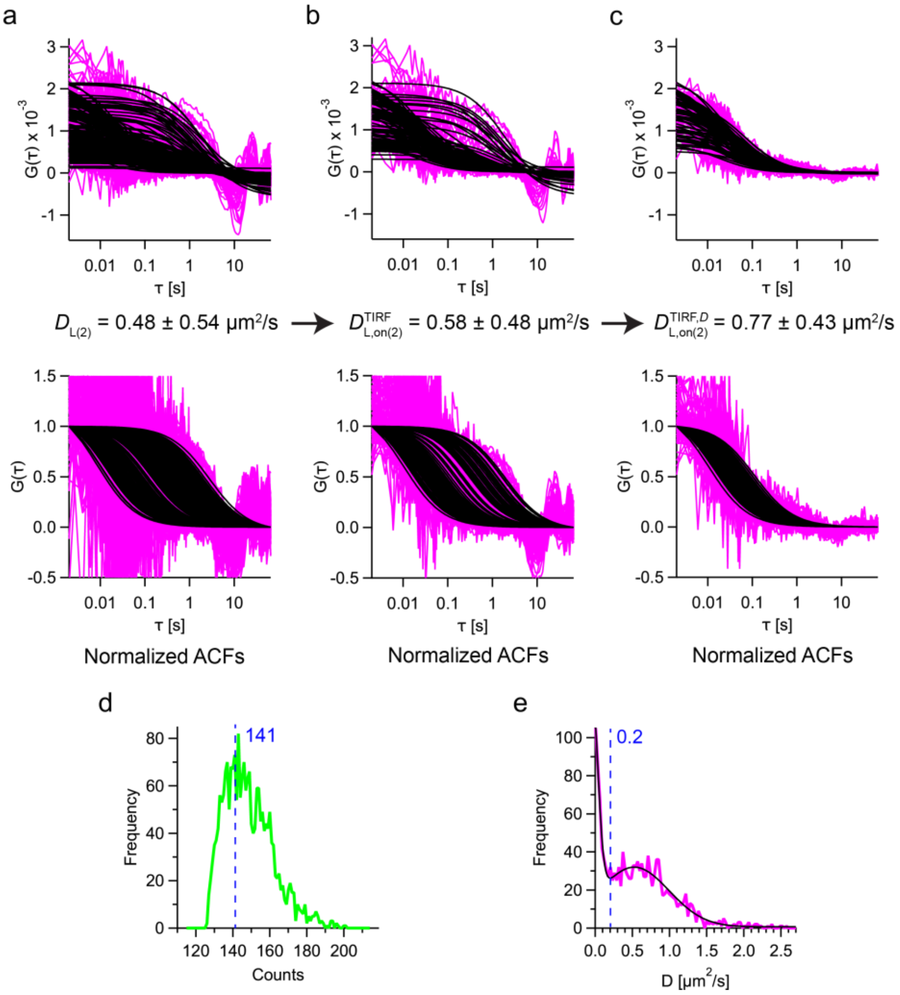

Supplementary Fig. 7: Normalized ACFs for the different thresholded *D* maps. (a) The ACFs (before and after normalization) for the *D* map without thresholding ($D_{\mathrm{L(2)}}$ = 0.48 ± 0.54 μm²/s) are shown. **(b)** The ACFs (before and after normalization) for the TIRF-masked *D* map ($D^{\mathrm{TIRF}}_{\mathrm{L,on(2)}}$ = 0.58 ± 0.48 μm²/s) are shown. **(c)** The ACFs (before and after normalization) for the *D* map after applying both TIRF mask and *D* thresholding ($D \geq 0.2$ μm²/s; $D^{\mathrm{TIRF},D}_{\mathrm{L,on(2)}}$ = 0.77 ± 0.43 μm²/s) are shown. Mean ± SD is reported here. **(d)** Intensity histogram showing the threshold used for creating the TIRF mask **(e)** The distribution of *D* showing the threshold used for filtering.

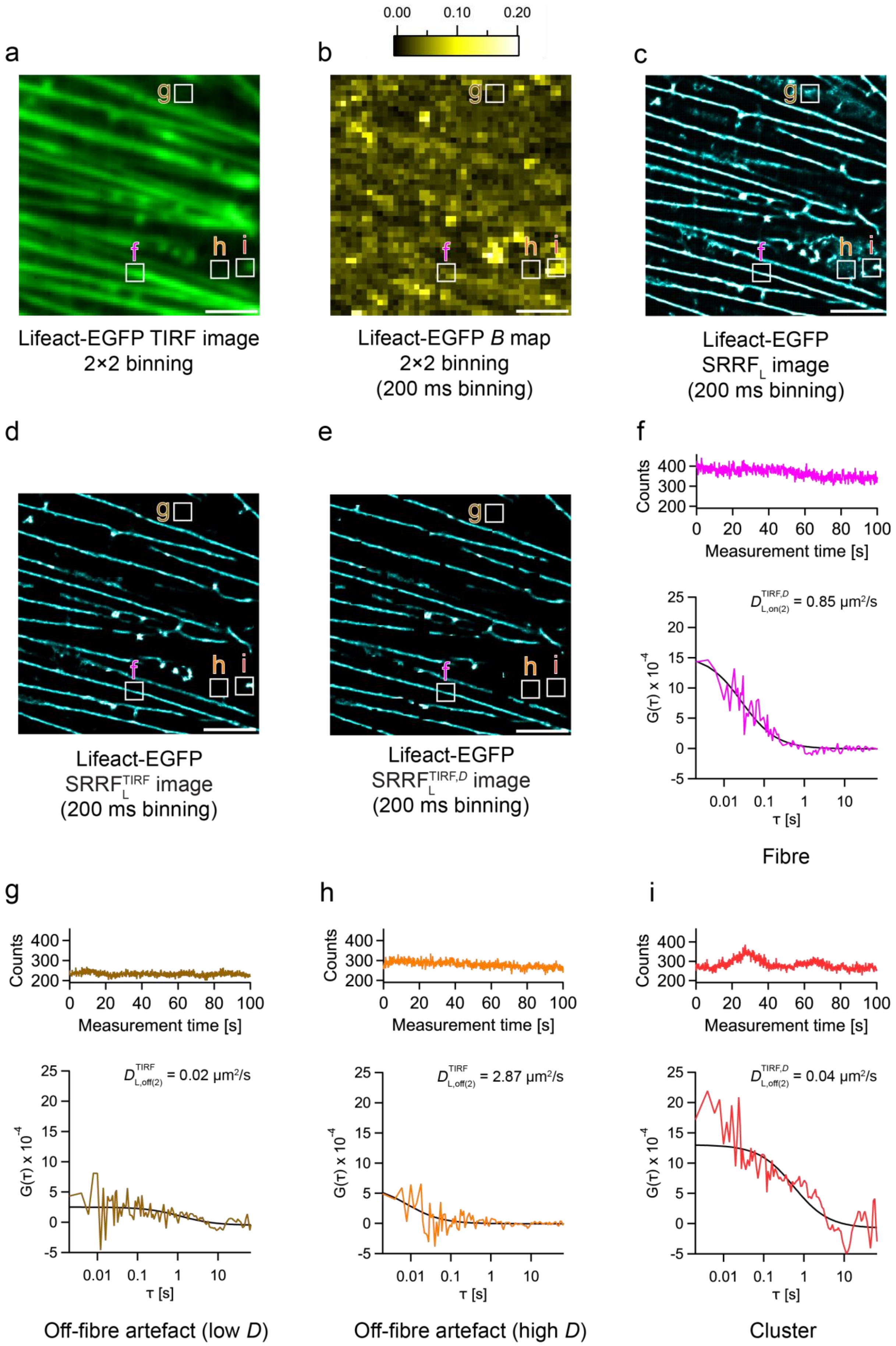
0.00
0.10
0.20
a
b
c
g
f
h
i
Lifeact-EGFP TIRF image
2×2 binning
Lifeact-EGFP B map
2×2 binning
(200 ms binning)
Lifeact-EGFP
SRRF_L image
(200 ms binning)
d
e
Lifeact-EGFP
SRRF_L^TIRF image
(200 ms binning)
Lifeact-EGFP
SRRF_L^TIRF,D image
(200 ms binning)
Counts
Measurement time [s]
G(τ) x 10^-4
τ [s]
D_L,on(2)^TIRF,D = 0.85 µm²/s
Fibre
D_L,off(2)^TIRF = 0.02 µm²/s
Off-fibre artefact (low D)
D_L,off(2)^TIRF = 2.87 µm²/s
Off-fibre artefact (high D)
D_L,off(2)^TIRF,D = 0.04 µm²/s
Cluster

Supplementary Fig. 8: SRRF artefacts give poor ACFs and one source of artefacts is clusters. The white boxes in images (a)-(e) show examples of fibre (f), off-fibre artefacts ((g), (h)), and cluster (i). **(a)** 2 × 2 pixel binned and time-averaged TIRF image of a CHO-K1 cell labelled with Lifeact-EGFP at ×200 magnification. **(b)** 2 × 2 pixel binned B map generated after time binning of the 2 ms data to 200 ms. Clusters can be seen as bright areas across the map. **(c)** Uncorrected $\mathrm{SRRF_L}$ image generated after time binning of the 2 ms data to 200 ms (Fig. 2f, Supplementary Fig. 4d). **(d)** $\mathrm{SRRF}_\mathrm{L}^\mathrm{TIRF}$ image **(e)** $\mathrm{SRRF}_\mathrm{L}^{\mathrm{TIRF},D}$ image. Panels (f)-(i) show the intensity traces across time (before bleach correction) and the ACFs (after bleach correction) for one pixel in each white box. **(f)** On a fibre, the intensity starts high and decreases with time due to bleaching. Upon fitting the bleach-corrected ACF, the diffusion coefficient is $D_{\mathrm{L,on(2)}}^{\mathrm{TIRF},D}$ =0.85 µm²/s. Fibres are retained in all 3 SRRF images (c)-(e). **(g)** In this off-fibre, cluster-free area, the intensity trace across time is flat with average counts below the TIRF mask threshold. The bleach-corrected ACF is noisy, has a very low amplitude and is fitted with a low *D* ($D_{\mathrm{L,off(2)}}^{\mathrm{TIRF}}$ = 0.02 µm²/s). **(h)** In another off-fibre, cluster-free area, the intensity trace across time shows some bleaching with average counts below the TIRF mask threshold. The bleach-corrected ACF is noisy, has a very low amplitude and is fitted with a high *D* ($D_{\mathrm{L,off(2)}}^{\mathrm{TIRF}}$= 2.87 µm²/s). Off-fibre artefacts like (g) and (h) are present in the uncorrected SRRF image (c), but are removed by the TIRF mask and are absent in the SRRF images (d) and (e). **(i)** In an off-fibre, cluster-containing area, the intensity trace shows intermittent broad peaks characteristic of a cluster. The average counts are above the TIRF mask threshold. The bleach-corrected ACF is noisy and does not fit properly to the one-component fitting model ($D_{\mathrm{L,off(2)}}^{\mathrm{TIRF},D}$ = 0.04 µm²/s). Clusters are present in the SRRF images (c) and (d) as they are not removed by the TIRF mask. They are absent in the $\mathrm{SRRF}_\mathrm{L}^{\mathrm{TIRF},D}$ image (e) as *D* thresholding removes them. The scale bars shown in white in (a)-(e) measures 2.5 µm. The analyses were performed on 4 cells from 4 different preparations of a single batch of cells with similar results. One representative cell is shown here.

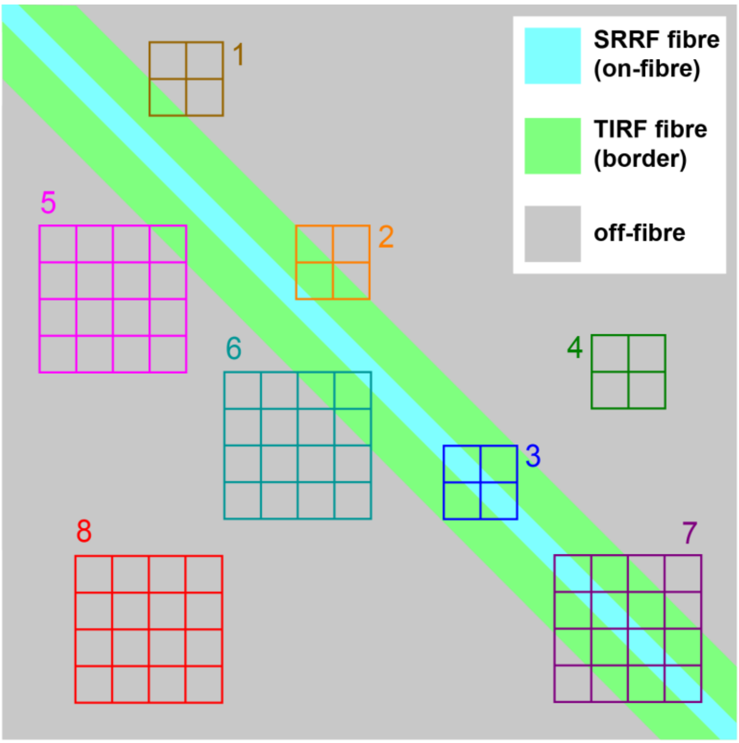

2×2 binning

4×4 binning (increased SNR; fast component fit only)

1: Border pixels → **5: Border pixels**

$D_{L,bd(2)}^{TIRF,D,SRRF}$ = 0.37 ± 0.45 μm²/s
COV = 122%

$D_{L,bd(4)}^{TIRF,D,SRRF}$ = 1.93 ± 1.69 μm²/s
COV = 88%

2: Super set of boxes 1 and 3 (Fig. 3) → **6: Super set of boxes 5 and 7**

$D_{L,on(2)}^{TIRF}$ = 0.58 ± 0.48 μm²/s
COV = 83%

$D_{L,on(4)}^{TIRF}$ = 1.47 ± 0.99 μm²/s
COV = 67%

3: on-fibre → **7: on-fibre (Supplementary Fig. 10)**

$D_{L,on(2)}^{TIRF,D,SRRF}$ = 0.80 ± 0.41 μm²/s
COV = 51%

$D_{L,on(4)}^{TIRF,D,SRRF}$ = 1.37 ± 0.73 μm²/s
COV = 53%

4: off-fibre (Supplementary Fig. 10) → **8: off-fibre (Supplementary Fig. 10)**

Low SNR

$D_{L,off(4)}^{TIRF}$ = 4.02 ± 3.98 μm²/s
COV = 99%

Supplementary Fig. 9: Dynamics of Lifeact at different length scales probed by SRRF and TIRF microscopy: The boxes 1-4 and 5-8 represent different categories of areas varying in proportions of fibre and off-fibre contributions at 2 × 2 and 4 × 4 binning, respectively. Boxes 2 and 6 are super sets of boxes 1&3 and boxes 5&7 respectively. For representative ACFs of each box refer Supplementary Fig. 11. All values are reported as Mean ± SD. We refer to pixels that are found to be on fibres in the $\mathrm{SRRF}_{\mathrm{L}}^{\mathrm{TIRF},D}$ image as "on-fibre". The pixels present on fibres in the TIRF image but not on the $\mathrm{SRRF}_{\mathrm{L}}^{\mathrm{TIRF},D}$ image are referred to as "border pixels". The pixels which are not on fibres in the TIRF image are referred to as "off-fibre". Boxes with 2 × 2 and 4 × 4 binning use the same nomenclature but have different contributions from molecules diffusing *on fibres* and *off fibres*.

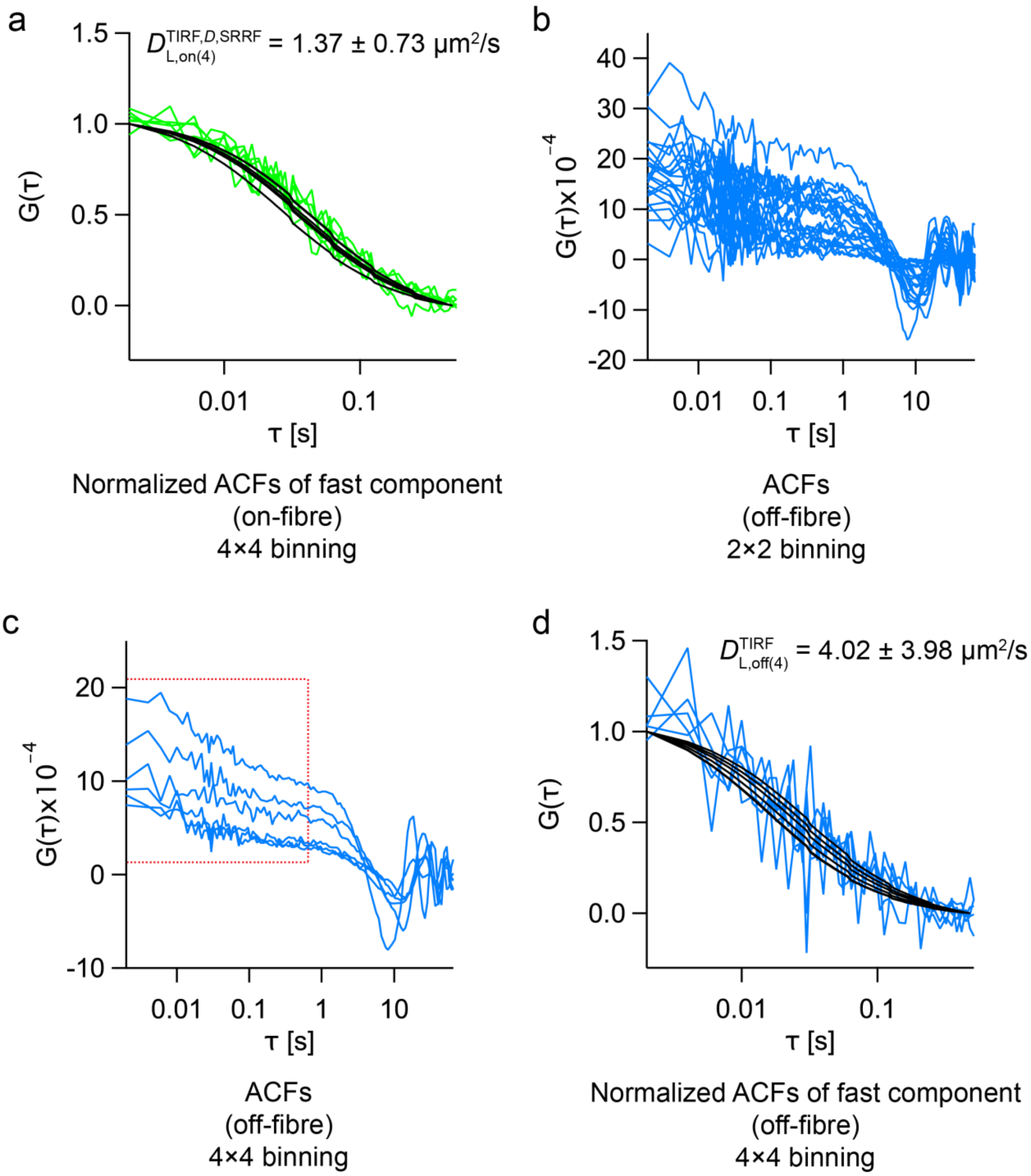

Supplementary Fig. 10: Off-fibre areas show a fast diffusion component: (a) Normalized ACFs of Lifeact-EGFP from fibre areas (on-fibre; Supplementary Fig. 9-box 7) after 4 × 4 binning at ×200 magnification ($D^{\text{TIRF},D,\text{SRRF}}_{\text{L,on(4)}}$ = 1.37 ± 0.73 μm²/s). **(b)** ACFs of Lifeact-EGFP from off-fibre areas (Supplementary Fig. 9-box 4) after 2×2 binning at ×200 magnification. **(c)** ACFs of Lifeact-EGFP from the same areas in (b) after 4 × 4 binning (Supplementary Fig. 9-box 8) at ×200 magnification. The fast component is marked with a red box. **(d)** Normalized ACFs of the fast diffusion component ($D^{\text{TIRF}}_{\text{L,off(4)}}$ = 4.02 ± 3.98 μm²/s). Mean ± SD is reported here.

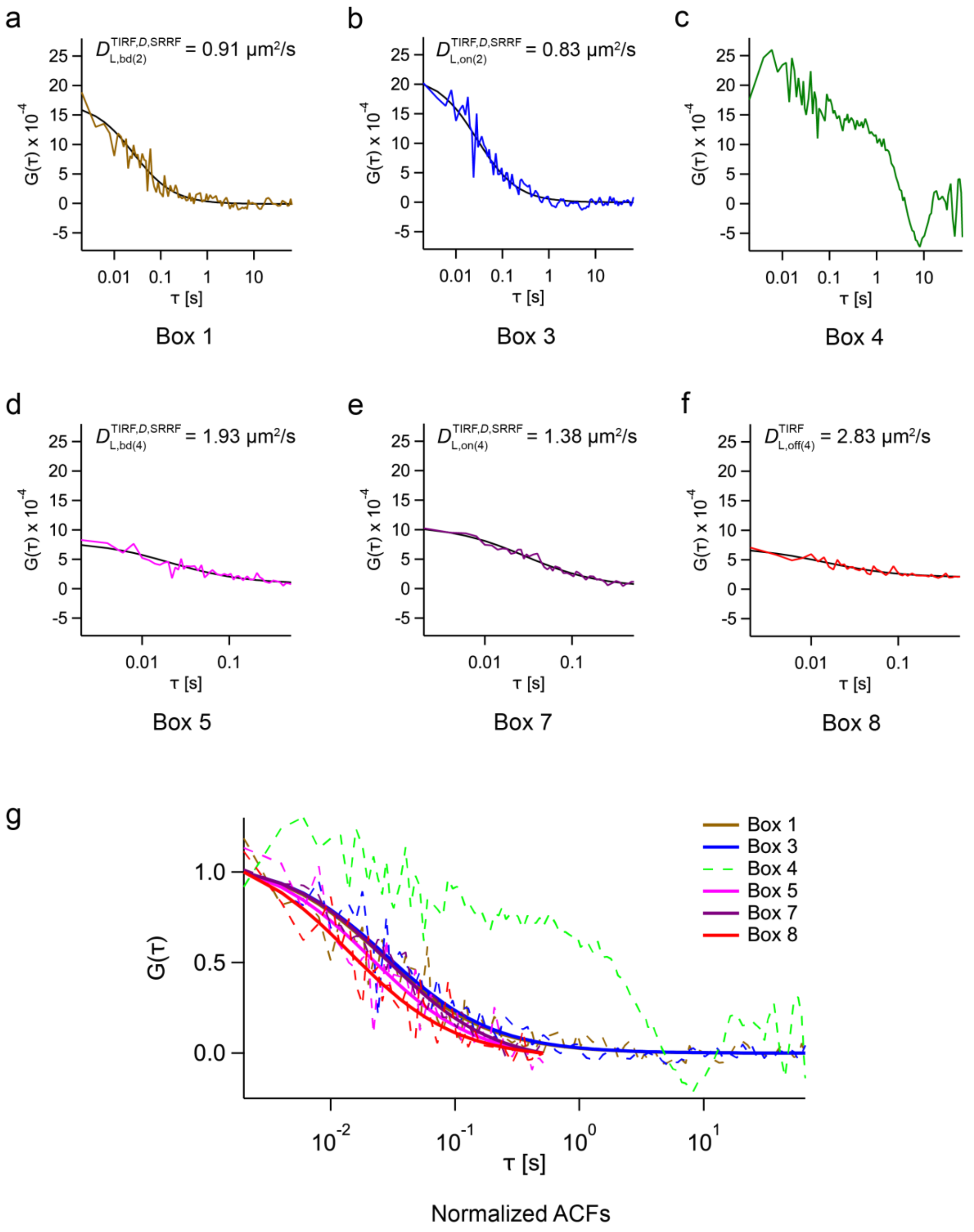

Supplementary Fig. 11: Representative ACFs from fibre and off-fibre areas. This figure shows the representative ACFs for the different colored boxes shown in the schematic of Supplementary Fig. 9. The data from boxes 1,3 and 4 are from 2 × 2 binning and fitted for the full measurement time of 100 seconds. The data from boxes 5, 7 and 8 are from 4 × 4 binning and only the fast *D* is fitted. **(a)** Representative ACF from box 1 (border pixels; $D_{\mathrm{L,bd(2)}}^{\mathrm{TIRF},D,\mathrm{SRRF}}$ = 0.91 µm²/s). **(b)** Representative ACF from box 3 (on-fibre; $D_{\mathrm{L,on(2)}}^{\mathrm{TIRF},D,\mathrm{SRRF}}$ = 0.83 µm²/s). **(c)** Representative ACF from box 4 (off-fibre; low SNR). **(d)** Representative ACF from box 5 (border pixels; $D_{\mathrm{L,bd(4)}}^{\mathrm{TIRF},D,\mathrm{SRRF}}$ = 1.93 µm²/s). **(e)** Representative ACF from box 7 (on-fibre; $D_{\mathrm{L,on(4)}}^{\mathrm{TIRF},D,\mathrm{SRRF}}$= 1.38 µm²/s). **(f)** Representative ACF from box 8 (off-fibre; $D_{\mathrm{L,off(4)}}^{\mathrm{TIRF}}$ = 2.83 µm²/s). **(g)** Normalized representation of all ACFs shown in (a)-(f).

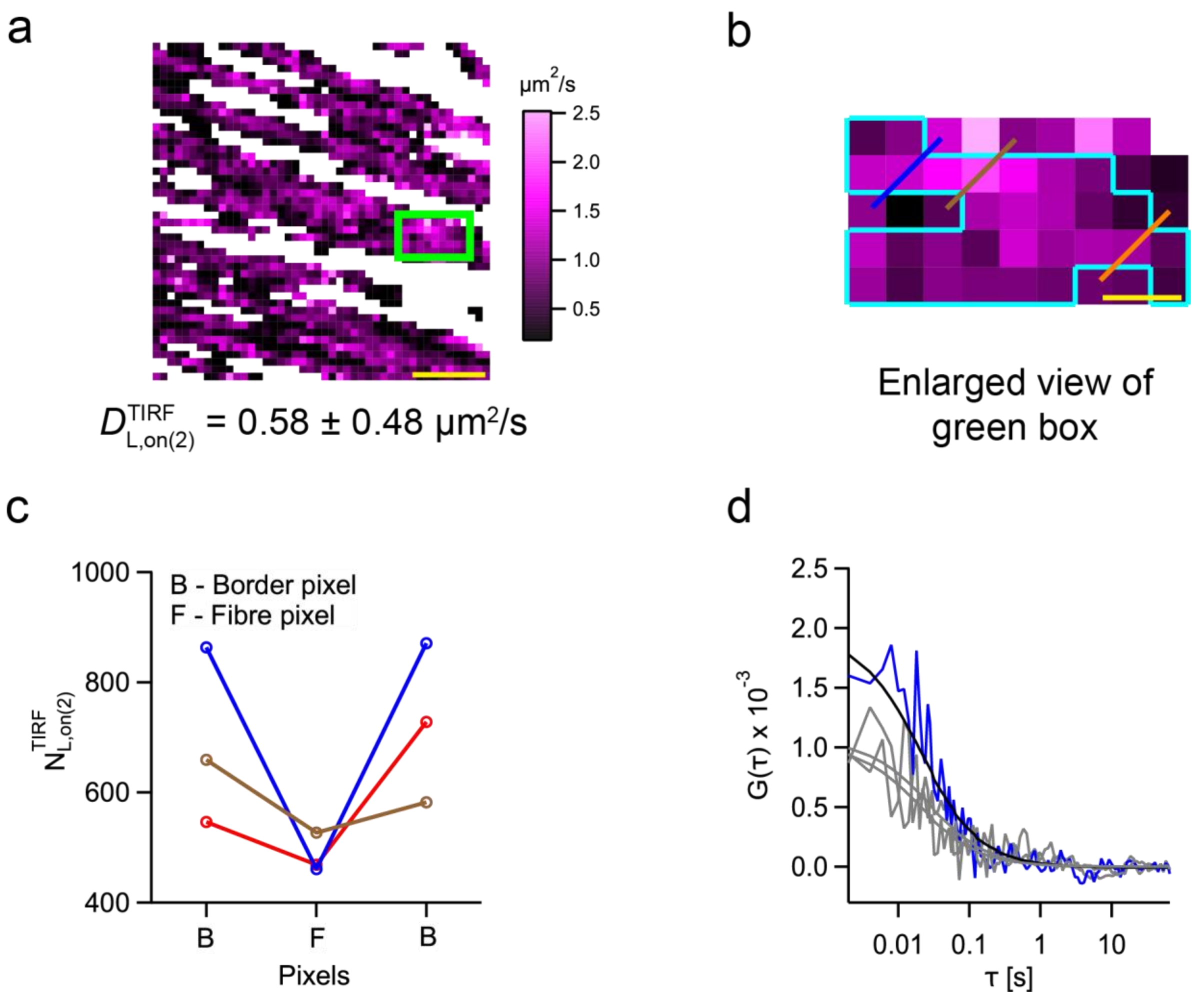

Supplementary Fig. 12: Autocorrelation functions of pixels located on SRRF map: (a) The D_{TIRF} map ($D^{TIRF}_{L,on(2)}$ = 0.58 ± 0.48 μm²/s) is shown at the top. The COV is 83%. **(b)** The enlarged view of the area labelled with the green box is shown below. The pixels bounded by the cyan lines are the pixels that overlap with SRRF image (white pixels in Fig. 2g). **(c)** The plot of $N^{TIRF}_{L,on(2)}$ versus the pixel type (F: fibre pixel, B: border pixel) for the three colored lines drawn in (b). Each colored line covers 3 pixels – a central pixel (on-fibre; Supplementary Fig. 9-box 3) flanked by two border pixels (Supplementary Fig. 9-box 1). All pixels are plotted from the left to right for each line. **(d)** The ACFs of the blue line in (b). The ACF of the on-fibre pixel (pixel F in (c)) is shown in blue, while the ACFs of the border pixels are shown in grey. The scale bars shown in yellow measure 2.5 μm in images (a) and 250 nm in (b) respectively. Mean ± SD of the *D* values are reported here. The analyses were performed on 4 cells from 4 different preparations of a single batch of cells with similar results (Supplementary Table 5). One representative cell is shown here.

Supplementary Table 5: FCS parameters *on* and *off* fibres

Cell	Diffusion Coefficient	Bin = 2		Bin = 4		Number of Particles	Bin = 2		Bin = 4		Number of Pixels	Bin = 2	Bin = 4
		Mean ± SD µm²/s	% COV	Mean ± SD µm²/s	% COV		Mean ± SD per pixel	% COV	Mean ± SD per pixel	% COV			
1	$D_{\mathrm{L(Bin)}}$	0.48 ± 0.54	113	1.83 ± 1.96	107	$N_{\mathrm{L(Bin)}}$	1309 ± 1043	80	1405 ± 625	44	$n_{\mathrm{L(Bin)}}$	2115	527
	$D^{\mathrm{TIRF}}_{\mathrm{L,on(Bin)}}$	0.58 ± 0.48	83	1.47 ± 0.99	67	$N^{\mathrm{TIRF}}_{\mathrm{L,on(Bin)}}$	970 ± 521	54	1279 ± 452	35	$n^{\mathrm{TIRF}}_{\mathrm{L,on(Bin)}}$	1503	453
	$D^{\mathrm{TIRF},D,\mathrm{SRRF}}_{\mathrm{L,on(Bin)}}$	0.80 ± 0.41	51	1.37 ± 0.73	53	$N^{\mathrm{TIRF},D,\mathrm{SRRF}}_{\mathrm{L,on(Bin)}}$	687 ± 205	30	1168 ± 329	28	$n^{\mathrm{TIRF},D,\mathrm{SRRF}}_{\mathrm{L,on(Bin)}}$	760	372
	$D^{\mathrm{TIRF},D,\mathrm{SRRF}}_{\mathrm{L,bd(Bin)}}$	0.37 ± 0.45	122	1.93 ± 1.69	88	$N^{\mathrm{TIRF},D,\mathrm{SRRF}}_{\mathrm{L,bd(Bin)}}$	1259 ± 585	46	1791 ± 572	32	$n^{\mathrm{TIRF},D,\mathrm{SRRF}}_{\mathrm{L,bd(Bin)}}$	743	81
	$D^{\mathrm{TIRF}}_{\mathrm{L,off(Bin)}}$	NA		4.02 ± 3.98	99	$N^{\mathrm{TIRF}}_{\mathrm{L,off(Bin)}}$	NA		2178 ± 923	42	$n^{\mathrm{TIRF}}_{\mathrm{L,off(Bin)}}$	NA	74
2	$D_{\mathrm{L(Bin)}}$	0.48 ± 0.70	146	1.32 ± 1.11	84	$N_{\mathrm{L(Bin)}}$	1919 ± 1519	79	2493 ± 1260	51	$n_{\mathrm{L(Bin)}}$	1517	528
	$D^{\mathrm{TIRF}}_{\mathrm{L,on(Bin)}}$	0.62 ± 0.45	73	0.89 ± 0.42	47	$N^{\mathrm{TIRF}}_{\mathrm{L,on(Bin)}}$	1306 ± 528	40	2001 ± 613	31	$n^{\mathrm{TIRF}}_{\mathrm{L,on(Bin)}}$	1151	330
	$D^{\mathrm{TIRF},D,\mathrm{SRRF}}_{\mathrm{L,on(Bin)}}$	0.67 ± 0.42	63	0.86 ± 0.41	48	$N^{\mathrm{TIRF},D,\mathrm{SRRF}}_{\mathrm{L,on(Bin)}}$	1132 ± 316	28	1846 ± 438	24	$n^{\mathrm{TIRF},D,\mathrm{SRRF}}_{\mathrm{L,on(Bin)}}$	719	259
	$D^{\mathrm{TIRF},D,\mathrm{SRRF}}_{\mathrm{L,bd(Bin)}}$	0.53 ± 0.48	91	1.01 ± 0.44	44	$N^{\mathrm{TIRF},D,\mathrm{SRRF}}_{\mathrm{L,bd(Bin)}}$	1596 ± 666	42	2569 ± 802	31	$n^{\mathrm{TIRF},D,\mathrm{SRRF}}_{\mathrm{L,bd(Bin)}}$	432	71
	$D^{\mathrm{TIRF}}_{\mathrm{L,off(Bin)}}$	NA		2.02 ± 1.49	74	$N^{\mathrm{TIRF}}_{\mathrm{L,off(Bin)}}$	NA		3312 ± 1593	48	$n^{\mathrm{TIRF}}_{\mathrm{L,off(Bin)}}$	NA	198
3	$D_{\mathrm{L(Bin)}}$	0.17 ± 0.26	153	1.53 ± 0.92	60	$N_{\mathrm{L(Bin)}}$	2146 ± 1195	56	3595 ± 1497	42	$n_{\mathrm{L(Bin)}}$	2116	525
	$D^{\mathrm{TIRF}}_{\mathrm{L,on(Bin)}}$	0.25 ± 0.30	120	1.34 ± 0.64	48	$N^{\mathrm{TIRF}}_{\mathrm{L,on(Bin)}}$	2005 ± 866	43	3273 ± 1139	35	$n^{\mathrm{TIRF}}_{\mathrm{L,on(Bin)}}$	1332	398
	$D^{\mathrm{TIRF},D,\mathrm{SRRF}}_{\mathrm{L,on(Bin)}}$	0.49 ± 0.31	63	1.22 ± 0.48	39	$N^{\mathrm{TIRF},D,\mathrm{SRRF}}_{\mathrm{L,on(Bin)}}$	1823 ± 490	27	2909 ± 751	26	$n^{\mathrm{TIRF},D,\mathrm{SRRF}}_{\mathrm{L,on(Bin)}}$	402	201
	$D^{\mathrm{TIRF},D,\mathrm{SRRF}}_{\mathrm{L,bd(Bin)}}$	0.15 ± 0.24	160	1.47 ± 0.74	50	$N^{\mathrm{TIRF},D,\mathrm{SRRF}}_{\mathrm{L,bd(Bin)}}$	2082 ± 976	47	3644 ± 1334	37	$n^{\mathrm{TIRF},D,\mathrm{SRRF}}_{\mathrm{L,bd(Bin)}}$	930	197
	$D^{\mathrm{TIRF}}_{\mathrm{L,off(Bin)}}$	NA		2.13 ± 1.34	63	$N^{\mathrm{TIRF}}_{\mathrm{L,off(Bin)}}$	NA		4606 ± 1969	43	$n^{\mathrm{TIRF}}_{\mathrm{L,off(Bin)}}$	NA	127
4	$D_{\mathrm{L(Bin)}}$	0.24 ± 0.32	133	1.30 ± 0.77	59	$N_{\mathrm{L(Bin)}}$	1515 ± 679	45	1824 ± 539	30	$n_{\mathrm{L(Bin)}}$	1599	400
	$D^{\mathrm{TIRF}}_{\mathrm{L,on(Bin)}}$	0.26 ± 0.32	123	1.22 ± 0.63	52	$N^{\mathrm{TIRF}}_{\mathrm{L,on(Bin)}}$	1436 ± 589	41	1785 ± 493	28	$n^{\mathrm{TIRF}}_{\mathrm{L,on(Bin)}}$	1396	378
	$D^{\mathrm{TIRF},D,\mathrm{SRRF}}_{\mathrm{L,on(Bin)}}$	0.48 ± 0.31	65	1.05 ± 0.50	48	$N^{\mathrm{TIRF},D,\mathrm{SRRF}}_{\mathrm{L,on(Bin)}}$	1201 ± 313	26	1589 ± 300	19	$n^{\mathrm{TIRF},D,\mathrm{SRRF}}_{\mathrm{L,on(Bin)}}$	501	208
	$D^{\mathrm{TIRF},D,\mathrm{SRRF}}_{\mathrm{L,bd(Bin)}}$	0.14 ± 0.26	186	1.43 ± 0.72	50	$N^{\mathrm{TIRF},D,\mathrm{SRRF}}_{\mathrm{L,bd(Bin)}}$	1568 ± 662	42	2025 ± 571	28	$n^{\mathrm{TIRF},D,\mathrm{SRRF}}_{\mathrm{L,bd(Bin)}}$	895	170
	$D^{\mathrm{TIRF}}_{\mathrm{L,off(Bin)}}$	NA		2.64 ± 1.46	55	$N^{\mathrm{TIRF}}_{\mathrm{L,off(Bin)}}$	NA		2492 ± 820	33	$n^{\mathrm{TIRF}}_{\mathrm{L,off(Bin)}}$	NA	22

9 Supplementary Note 9: Estimating dimerization fraction of EGFR in live cells using N&B analysis

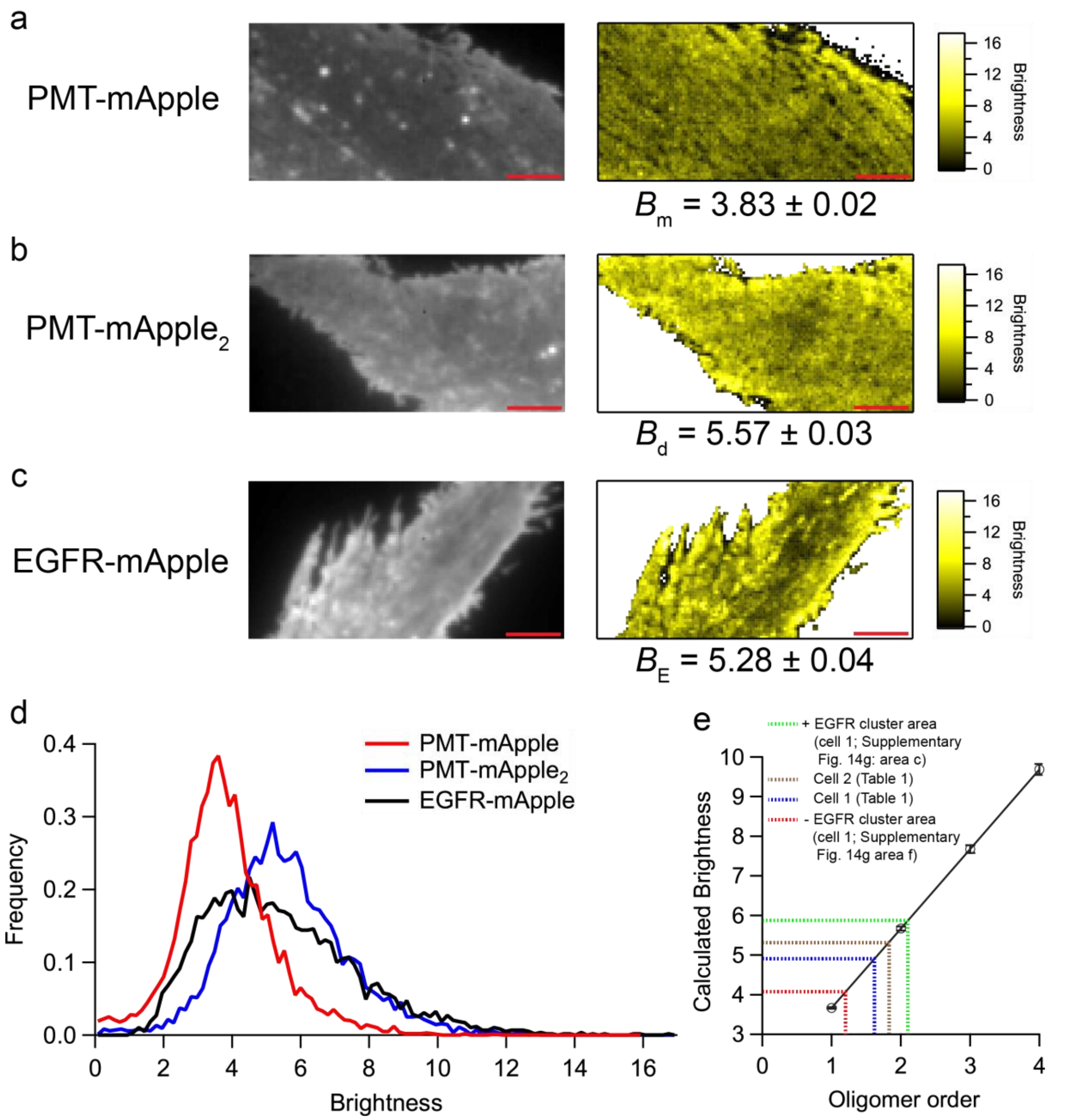

Supplementary Fig. 13: Number and Brightness analysis. (a) TIRF (left) and brightness (right) images of CHO-K1 cells expressing PMT-mApple, PMT-mApple$_2$ and EGFR-mApple (cell 2 in Table 1) are shown in **(a)**, **(b)** and **(c)**, respectively. The brightness histograms corresponding to the images shown in (a)-(c) are shown in **(d)**. **(e)** The scaling of expected brightness with oligomer order is shown. In the case of monomer and dimer, the brightness is experimentally determined (n = 3 cells). The trimer and tetramer values were determined theoretically (using Eq. 37). The SEM was calculated by error propagation (Eq. 39). The dotted lines indicate the average B_E values for different cases – an area with EGFR cluster in cell 1 (green; refer Supplementary Fig. 14g: area f), cell 2 (brown; refer Table 1), cell 1 (blue; refer Table 1) and an area without visible EGFR clusters in cell 1 (red; refer Supplementary Fig. 14g: area c). The scale bars shown in red measure 5 µm in images (a)-(c). The reported values in the figure are mean ± SEM. The analyses were performed on 3 cells from 3 different batches of cells for both PMT-mApple and PMT-mApple$_2$, and 6 cells from 3 different batches of cells for EGFR-mApple with similar results (Table 1). One representative cell for each case is shown here.

10 Supplementary Note 10: Diffusion law analysis of EGFR on live cells

We performed diffusion law analysis over different areas of CHO-K1 cells expressing EGFR-mApple to investigate the membrane organization. There were four kinds of areas which include the presence or absence of cytoskeleton or clusters.

Supplementary Table 6: Diffusion law intercepts in different areas of CHO-K1 cells labelled with EGFR-mApple*

**Cell**	**Diffusion law intercept [s]**			
	**+ Actin fibre<br>+ EGFR cluster**	**+ Actin fibre<br>- EGFR cluster**	**- Actin<br>+ EGFR cluster**	**- Actin fibre<br>- EGFR cluster**
1	2.56	2.01	1.81	1.81
2	2.53	2.42	3.43	-
3	2.21	1.48	-	0.40
4	5.3	2.4	4.47	3.16
5	2.02	1.37	-	1.22
6	2.17	2.06	1.73	-
**Mean**	**2.80 ± 1.24**	**1.96 ± 0.45**	**2.86 ± 1.33**	**1.65 ± 1.16**

*Please refer to Table 1 for other corresponding parameters of the cells. Some cells do not contain all types of areas, and thus no values are provided.

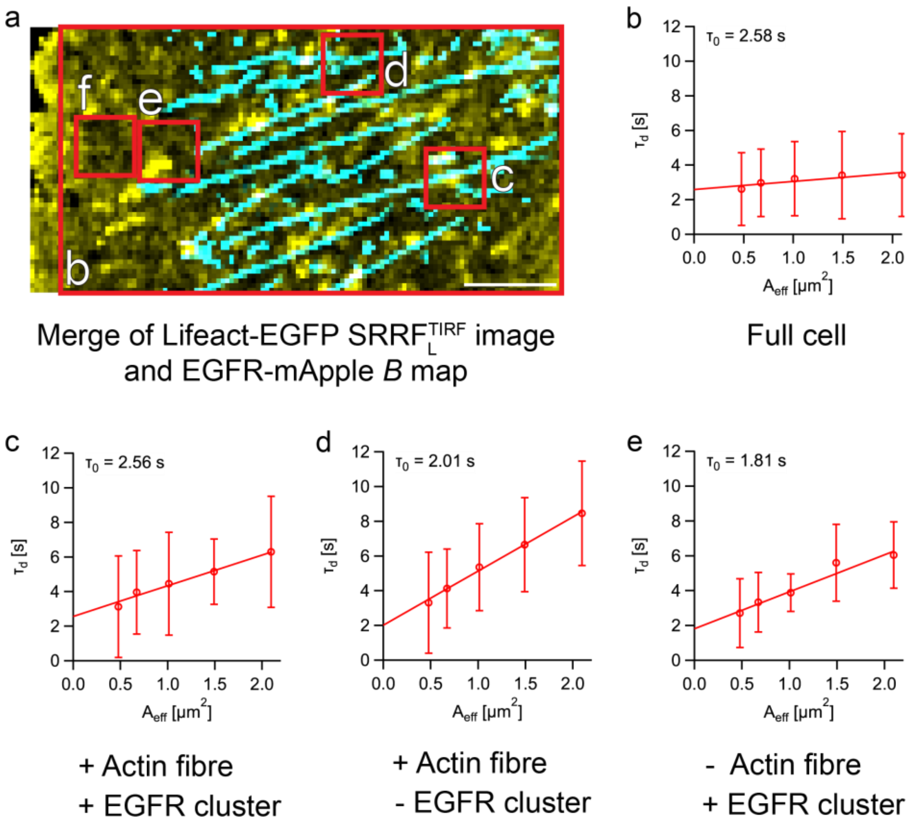

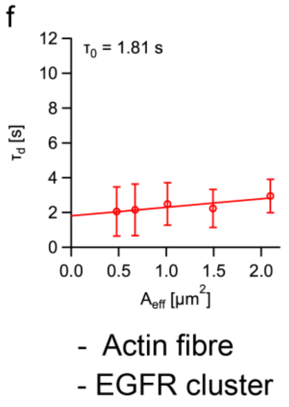

g

Area	D_E (μm²/s)	B_E (dimer fraction)	τ_0 (s)
b (114×56 pixels)	0.18 ± 0.15	4.80 ± 0.03 (0.57 ± 0.07)	2.58
c (10×10 pixels)	0.11 ± 0.08	5.87 ± 0.26 (NA)	2.56
d (10×10 pixels)	0.16 ± 0.14	4.15 ± 0.12 (0.24 ± 0.06)	2.01
e (10×10 pixels)	0.18 ± 0.13	7.55 ± 0.73 (NA)	1.81
f (10×10 pixels)	0.24 ± 0.16	4.03 ± 0.11 (0.18 ± 0.06)	1.81

Supplementary Fig. 14: Diffusion law analysis over different areas: (a) Merge of $\mathrm{SRRF}_L^{\mathrm{TIRF}}$ image and B_E map of a CHO-K1 cell expressing Lifeact-EGFP and EGFR-mApple (cell 1 from Table 1). The red boxes (numbered b-f) indicate the different areas tested. **(b)** Diffusion law plot in the area marked by the red box b (mean ± SD, *n* = 114 × 56 pixels). The intercept is 2.58 s. This is the largest rectangular box that could be fitted into the cell. **(c)** Diffusion law in area marked by the red box c (mean ± SD, *n* = 10 × 10 pixels). The intercept is 2.56 s. This area was chosen as there were both an EGFR cluster and an actin fibre. **(d)** Diffusion law in the area marked by the red box d (mean ± SD, *n* = 10 × 10 pixels). The intercept is 1.81 s. This area was chosen as there was neither an actin fibre nor an EGFR cluster. **(e)** Diffusion law in the area marked by the red box e (mean ± SD, *n* = 10 × 10 pixels). The intercept is 2.01 s. This area was chosen as there was an actin fibre but no EGFR cluster was present. **(f)** Diffusion law in the area marked by the red box f (mean ± SD, *n* = 10 × 10 pixels). The intercept is 1.81 s. This area was chosen as there was an EGFR cluster but no actin fibre was present. **(g)** Table of D_E (mean ± SD), B_E (dimer fraction in brackets; mean ± SEM), and intercept values in the chosen areas (b)-(f). For B_E higher than dimer control, the dimer model is not applicable and an oligomer model has to be used. The regions with cluster have a higher brightness when compared to those which do not have clusters as seen in Table g. The scale bar in red measures 5 µm in image (a). The analyses were performed on 6 cells from 3 different batches of cells with similar results (Supplementary Table 6).

11 Supplementary Note 11: Use of sCMOS in multi-parametric analysis

Here, we describe the optimization and results obtained using sCMOS as a detector for dual channel measurements on cells labelled with EGFR-mApple and Lifeact-EGFP. Since the pixels of the sCMOS camera are smaller than for the EMCCD, pixel binning is used to optimize the FCS signal. Here we used 2 × 2 binning on data sets acquired at 2 ms using sCMOS. In the case of diffusion law analysis, it was calculated from 3 × 3 to 7 × 7 binning. N&B was performed with 2 × 2 spatial binning at 20 ms time binning using 5,000 frames.

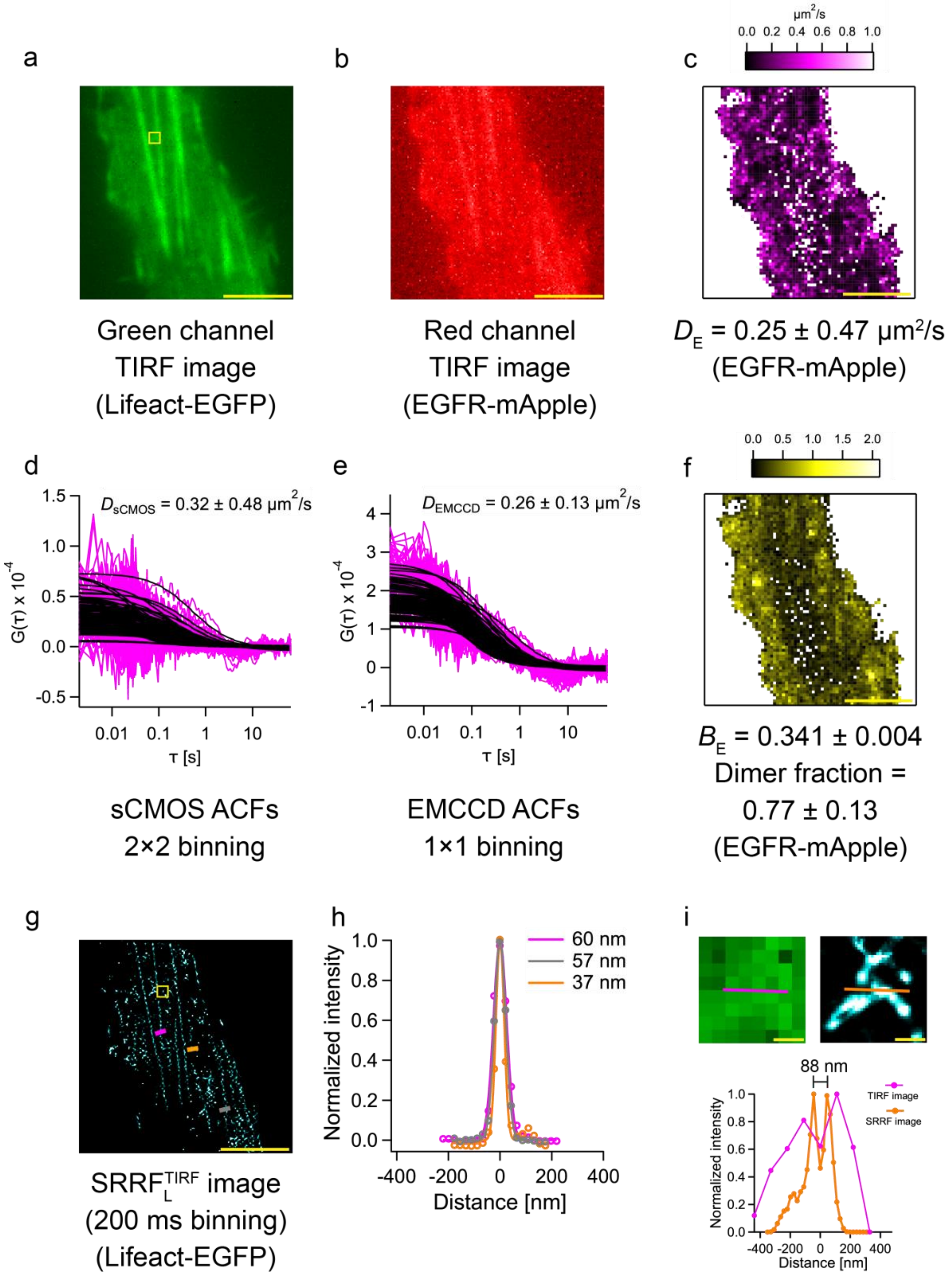

Supplementary Fig. 15: Multi-parametric analysis using sCMOS: (a) and **(b)** are TIRF images of CHO-K1 cells labelled with Lifeact-EGFP and EGFR-mApple. **(c)** Diffusion map of EGFR-mApple. **(d)** and **(e)** show ACFs for EGFR-mApple from sCMOS and EMCCD, respectively. The sCMOS (magnified pixel size = 110 nm) data was binned 2 × 2 (chosen area = 2.2 µm × 2.2 µm), while the EMCCD (magnified pixel size = 240 nm) data was not spatially binned (chosen area = 2.4 µm × 2.4 µm). The mean and standard deviation of the diffusion coefficient estimated only from the displayed curves is shown. **(f)** Brightness map of EGFR-mApple (B_E = 0.341 ± 0.004, dimer fraction-m_e = 0.77 ± 0.13; mean ± SEM). **(g)** $\mathrm{SRRF}_{\mathrm{L}}^{\mathrm{TIRF}}$ image (200 ms binning) of Lifeact EGFP. **(h)** Normalized intensity profile across actin fibres (*n* = 3 positions) on the cell (e). All reported values in the figure are the FWHM of the Gaussian fits (average = 51 ± 10 nm). **(i)** Enlarged views of the yellow boxes in images (a) and (g). The intensity profile below shows the actin fibre branching point (indicated by the orange line on the enlarged SRRF image). The peak-to-peak resolution is 88 nm at this point. The scale bars shown in yellow in (a,b,c,f,g) measure 5 µm, and 250 nm in (i). Unless otherwise stated, all values are given as mean ± SD. The analyses were performed on 3 cells from 3 different batches of cells with similar results.

The estimated D_E measured using a sCMOS is 0.25 ± 0.47 µm^2/s. This corresponds to a COV of 188%. The COV of D_E using an EMCCD is 79% (0.19 ± 0.15 µm^2/s, Fig. 4, Supplementary Fig. 15e). The COV of sCMOS is larger than for the EMCCD due to a lower SNR of the obtained ACFs using a sCMOS (Supplementary Fig. 15). It is important to note that the inclusion of an optosplit in the detection path for two channel measurements leads to additional losses in collected fluorescence signal. The reduction in collected signal intensity leads to lower SNR of ACFs. In the case of measurements with sCMOS, the dimer fraction (m_e) is found to be 77 ± 13%. The larger error for the dimer fraction of EGFR using the sCMOS camera is also attributed to the reduced SNR (Supplementary Fig. 15). In the case of SRRF image obtained after TIRF masking, a FWHM of 51 ± 10 nm is obtained using 200 ms binning. The mean FRC was 63 ± 11 nm while the P2P was 88 ± 22 nm (Supplementary Fig. 15).

The diffusion law analysis performed with the sCMOS camera is shown in Supplementary Fig. 16. The intercept values are similar to those obtained using the EMCCD (Supplementary Table 6, Supplementary Fig. 14).

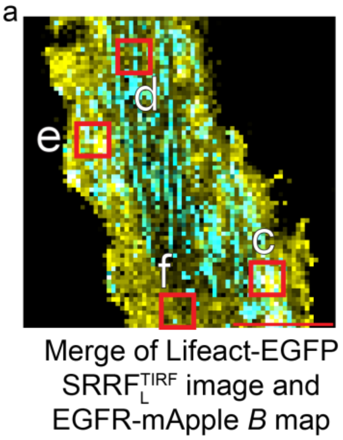

b

Area	D_E (μm²/s)	B_E (dimer fraction)	T_0 (s)
c (14×14 pixels)	0.14 ± 0.16	0.58 ± 0.04 (NA)	2.78
d (14×14 pixels)	0.23 ± 0.20	0.27 ± 0.02 (0.20 ± 0.20)	1.01
e (14×14 pixels)	0.19 ± 0.24	0.63 ± 0.06 (NA)	1.00
f (14×14 pixels)	0.29 ± 0.27	0.28 ± 0.02 (0.29 ± 0.21)	0.88

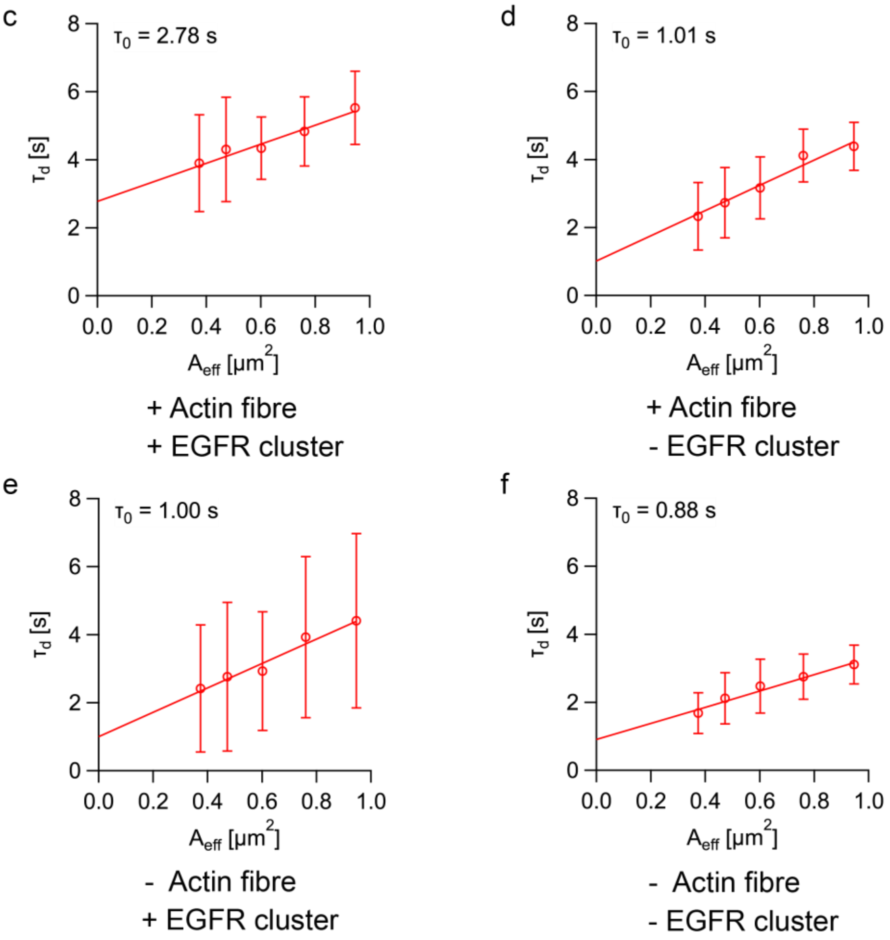

Supplementary Fig. 16: Diffusion law analysis over different areas of a CHO-K1 cell expressing EGFR-mApple cell acquired using a sCMOS: (a) Merge of $\mathrm{SRRF}_L^{\mathrm{TIRF}}$ image and B_E map of a CHO-K1 cell expressing Lifeact-EGFP and EGFR-mApple. The red boxes (numbered c-f) indicate the different areas tested. **(b)** Table of D_E (mean ± SD), B_E (mean ± SEM) and intercept values in the chosen areas c-f. The regions with clusters have a higher brightness when compared to regions without clusters. **(c)** Diffusion law in area marked by the red box c (mean ± SD, *n* = 14 × 14 pixels). The intercept is 2.78 s. This area was chosen as both an EGFR cluster and an actin fibre were present. **(d)** Diffusion law in area marked by the red box d (mean ± SD, *n* = 14 × 14 pixels). The intercept is 1.01 s. This area was chosen as there was an EGFR cluster but no actin fibre present. **(e)** Diffusion law in area marked by the red box e (mean ± SD, *n* = 14 × 14 pixels). The intercept is 1.00 s. This area was chosen as there was an actin fibre but no EGFR cluster present. **(f)** Diffusion law in area marked by the red box f (mean ± SD, *n* = 14 × 14 pixels). The intercept is 0.88 s. This area was chosen as there was neither an actin fibre nor an EGFR cluster. The scale bar in red measures 5 μm in image (a). The analyses were performed on 3 cells from 3 different batches of cells with similar results.

12 Supplementary Note 12: Theoretical estimation of maximum brightness and brightness ratio of cells

In the case of mApple, the proportion of molecules that were found to be fluorescent was 55% and the brightness of the monomers were estimated to be 3.67 ± 0.02. The theoretically estimated maximum brightness of pure population of oligomers and maximum ratio of brightness of oligomer to monomer are tabulated here. The error associated with each of the estimates were obtained by performing an error propagation on the errors associated with proportion of fluorescent molecule and the average brightness of the monomers (section 3.4.1).

Supplementary Table 7: Theoretical estimation of maximum brightness and brightness ratio of cells expressing mApple oligomers using experimentally determined proportion of fluorescent molecules of 55%

Type	Order of oligomer	Maximum ratio of brightness of oligomer to monomer	Maximum brightness of pure population of oligomers
**Experimental**	1	1	3.67 ± 0.02
**Estimated**	2	1.55 ± 0.01	5.67 ± 0.05
	3	2.09 ± 0.02	7.68 ± 0.10
	4	2.64 ± 0.04	9.68 ± 0.14
	5	3.19 ± 0.05	11.69 ± 0.18
	6	3.74 ± 0.06	13.70 ± 0.23
	7	4.28 ± 0.07	15.70 ± 0.27
	8	4.83 ± 0.08	17.71 ± 0.32
	9	5.38 ± 0.10	19.72 ± 0.36
	10	5.93 ± 0.11	21.72 ± 0.41

13 Supplementary figures for "Modulation of EGFR dynamics and clustering"

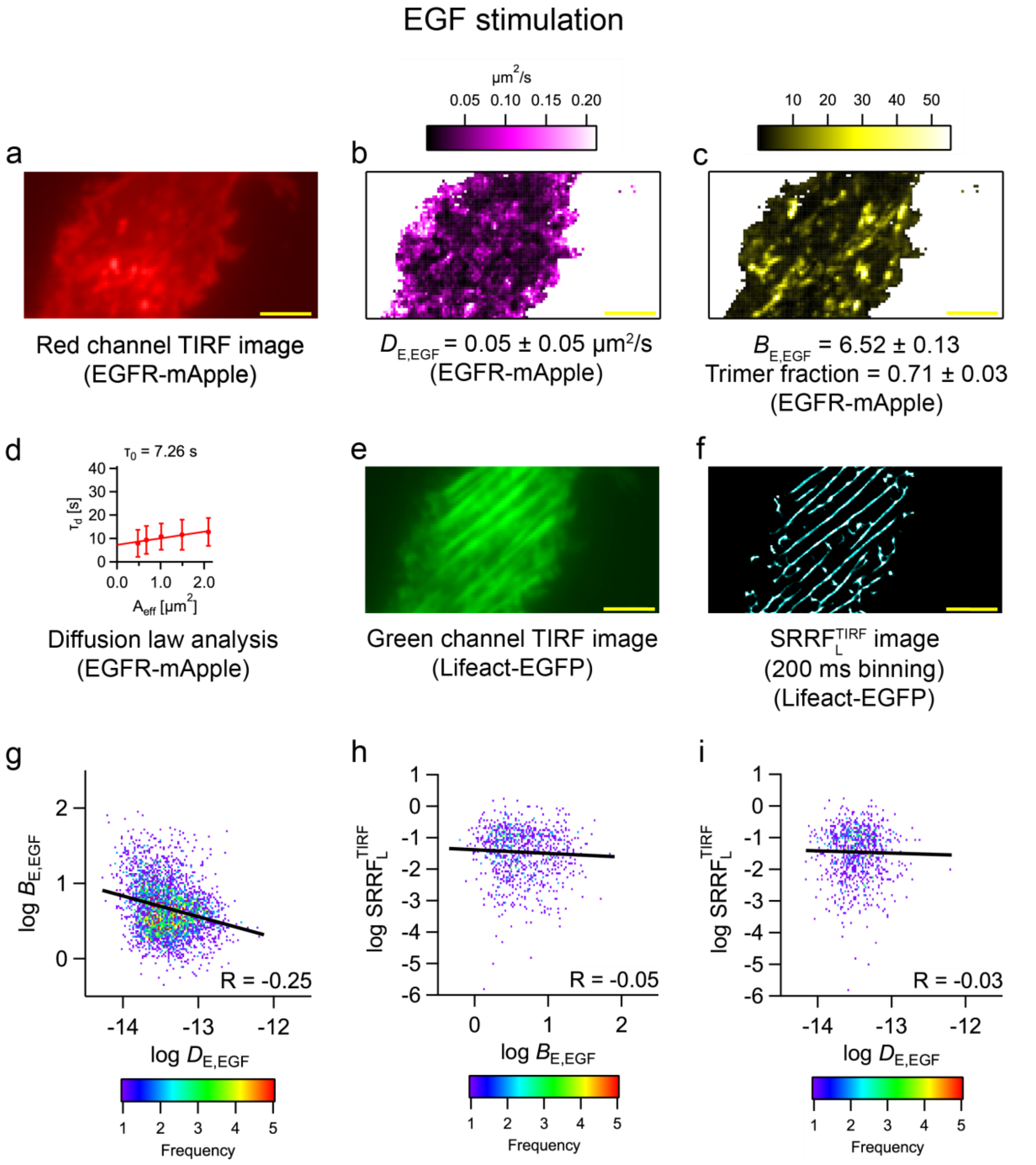

Supplementary Fig. 17: Multi-parametric analysis after 100 ng/ml EGF stimulation: a) TIRF image of EGFR-mApple. **(b)** Diffusion map of EGFR-mApple ($D_{E,EGF}$ = 0.05 ± 0.05 µm²/s). **(c)** Brightness map of EGFR-mApple ($B_{E,EGF}$ = 6.52 ± 0.12, trimer fraction-e_{trimer} = 0.71 ± 0.04; mean ± SEM). The brightness values here are expressed in terms of the maximum oligomer fraction for the minimum required oligomeric state. **(d)** Diffusion law analysis of EGFR-mApple; intercept = 7.26 s; values are mean ± SD (n = 1600 pixels at 1 × 1 binning). **(e)** TIRF image of Lifeact-EGFP. **(f)** $\mathrm{SRRF}_{\mathrm{L}}^{\mathrm{TIRF}}$ image (200 ms binning) of Lifeact-EGFP. **(g)** 2D frequency plot of log $B_{E,EGF}$ vs log $D_{E,EGF}$ values. **(h)** 2D frequency plot of log $\mathrm{SRRF}_{\mathrm{L}}^{\mathrm{TIRF}}$ vs log $B_{E,EGF}$. **(i)** 2D frequency plot of log $\mathrm{SRRF}_{\mathrm{L}}^{\mathrm{TIRF}}$ vs log $D_{E,EGF}$ values. The scale bars in yellow measure 5 µm in images (a)-(c), (e) and (f). This figure uses cell 1 in Table 2. The reported values are averages obtained from analysis of an entire cell. The analyses were performed on 3 cells from 3 different batches of cells with similar results (Table 2).

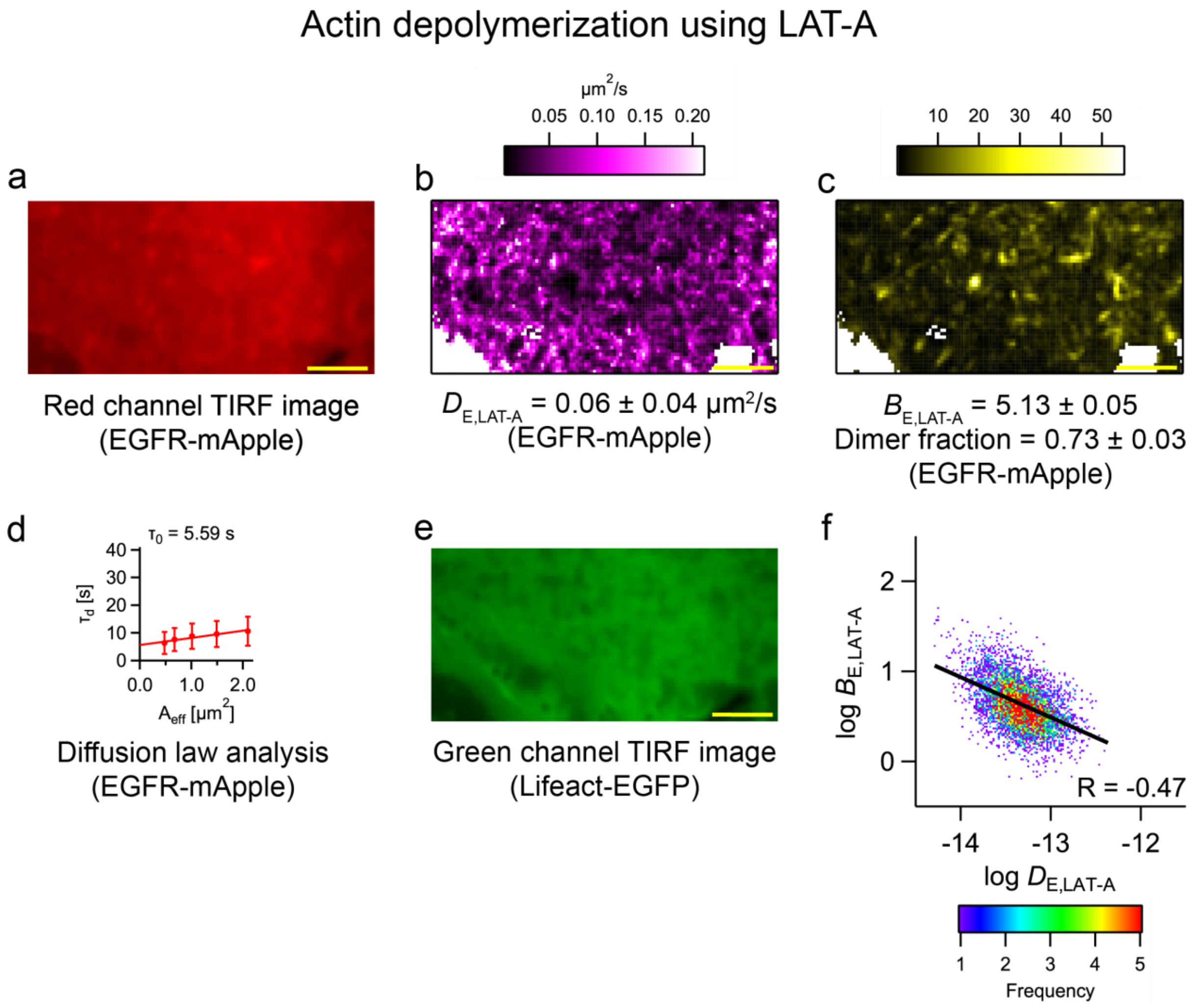

Supplementary Fig. 18: Multi-parametric analysis 3 µM LAT-A treatment. (a) TIRF image of EGFR-mApple. **(b)** Diffusion map of EGFR-mApple ($D_{E,LAT\text{-}A}$ = 0.06 ± 0.04 µm^2/s). **(b)** Brightness map of EGFR-mApple ($B_{E,LAT\text{-}A}$ = 5.13 ± 0.05, dimer fraction-m_e = 0.73 ± 0.03; mean ± SEM). **(d)** Diffusion law analysis of EGFR-mApple; intercept = 5.59 s; values are mean ± SD (n = 3000 pixels at 1 × 1 binning). **(e)** TIRF image of Lifeact-EGFP. **(f)** 2D frequency plot of log $B_{E,LAT\text{-}A}$ vs log $D_{E,LAT\text{-}A}$ values. The scale bars in yellow measure 5 µm in images (a)-(c), (e). This figure uses cell 1 in Table 2. The reported values are averages obtained from analysis of an entire cell. The analyses were performed on 3 cells from 3 different batches of cells with similar results (Table 2).

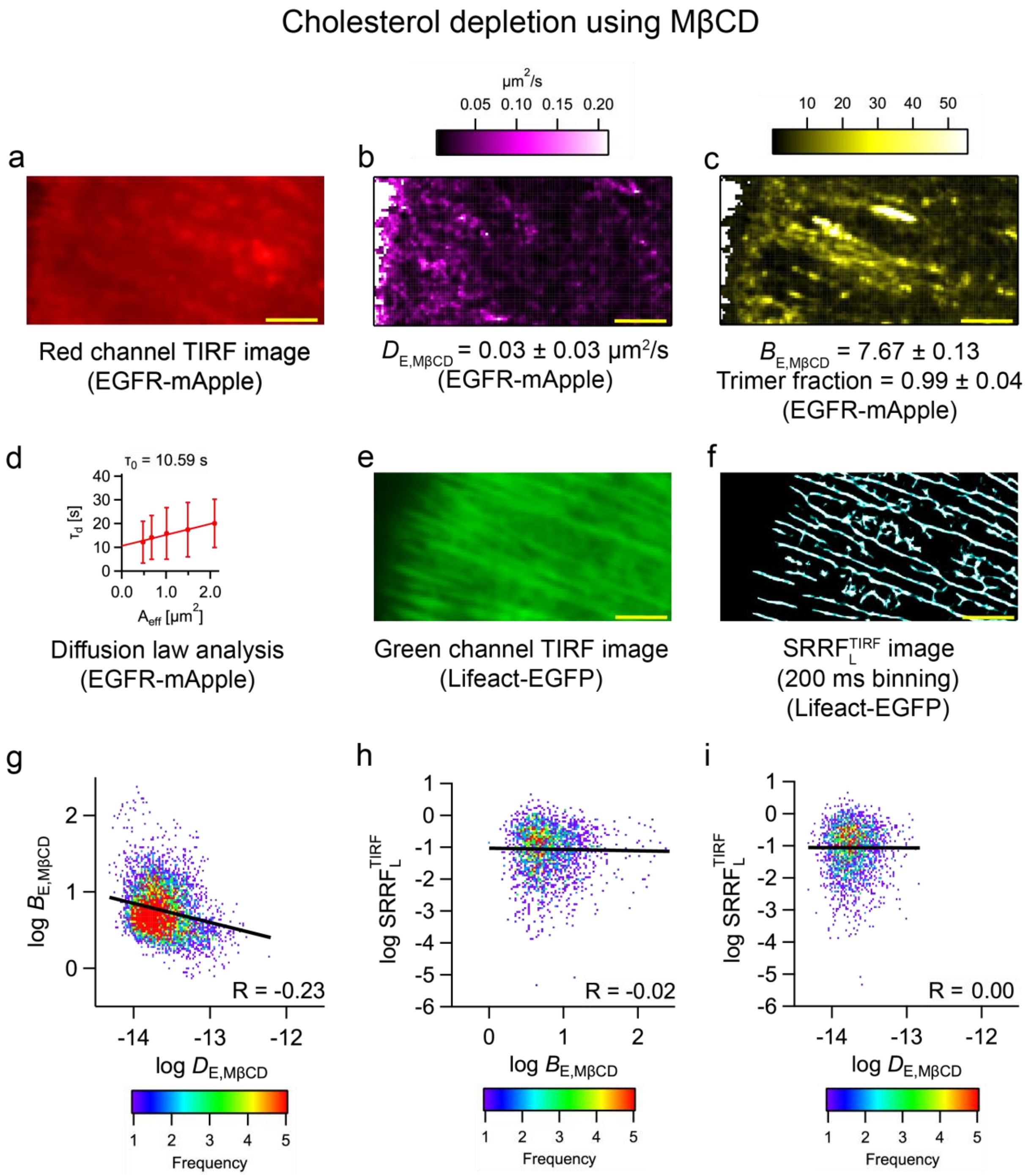

Supplementary Fig. 19: Multi-parametric analysis after cholesterol depletion using 3 mM MβCD: **(a)** TIRF image of EGFR-mApple. **(b)** Diffusion map of EGFR-mApple ($D_{E,M\beta CD}$ = 0.03 ± 0.03 μm^2/s). **(c)** Brightness map of EGFR-mApple ($B_{E,M\beta CD}$ = 7.67 ± 0.13, trimer fraction-e_{trimer} = 0.99 ± 0.04; mean ± SEM). **(d)** Diffusion law analysis of EGFR-mApple; intercept = 10.59 s; values are mean ± SD (n = 6160 pixels at 1 × 1 binning). **(e)** TIRF image of Lifeact-EGFP. **(f)** $\mathrm{SRRF}_{\mathrm{L}}^{\mathrm{TIRF}}$ image (200 ms binning) of Lifeact-EGFP. **(g)** 2D frequency plot of log $B_{E,M\beta CD}$ vs log $D_{E,M\beta CD}$ values. **(h)** 2D frequency plot of log $\mathrm{SRRF}_{\mathrm{L}}^{\mathrm{TIRF}}$ vs log $B_{E,M\beta CD}$. **(i)** 2D frequency plot of log $\mathrm{SRRF}_{\mathrm{L}}^{\mathrm{TIRF}}$ vs log $D_{E,M\beta CD}$ values. The scale bars in yellow measure 5 μm in images (a)-(c), (e) and (f). This figure uses cell 1 in Table 2. The reported values are averages obtained from analysis of an entire cell. The analyses were performed on 3 cells from 3 different batches of cells with similar results (Table 2).

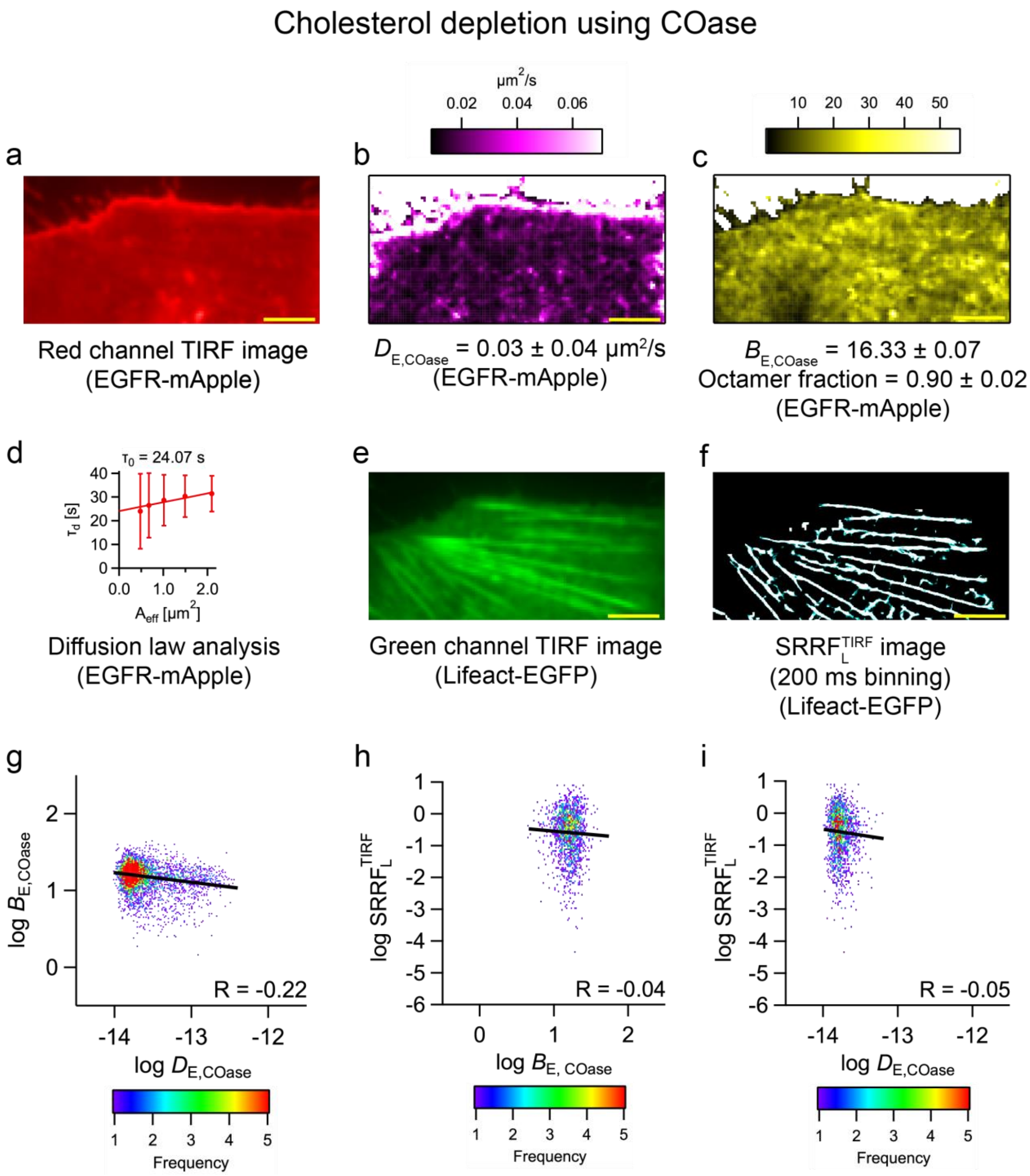

Supplementary Fig. 20: Multi-parametric analysis after cholesterol depletion using 1 U/ml COase. (a) TIRF image of EGFR-mApple. **(b)** Diffusion map of EGFR-mApple ($D_{\mathrm{E,COase}}$ = 0.03 ± 0.04 $\mu m^2/s$). **(c)** Brightness map of EGFR-mApple ($B_{\mathrm{E,COase}}$ = 16.33 ± 0.07, octamer fraction-$e_{octamer}$ = 0.90 ± 0.02; mean ± SEM). **(d)** Diffusion law analysis of EGFR-mApple; intercept = 24.07 s; values are mean ± SD (n = 3960 pixels at 1 × 1 binning). **(e)** TIRF image of Lifeact-EGFP. **(f)** $\mathrm{SRRF}_{\mathrm{L}}^{\mathrm{TIRF}}$ image (200 ms binning) of Lifeact-EGFP. **(g)** 2D frequency plot of log $B_{\mathrm{E,COase}}$ vs log $D_{\mathrm{E,COase}}$ values. **(h)** 2D frequency plot of log $\mathrm{SRRF}_{\mathrm{L}}^{\mathrm{TIRF}}$ vs log $B_{\mathrm{E,COase}}$. **(i)** 2D frequency plot of log $\mathrm{SRRF}_{\mathrm{L}}^{\mathrm{TIRF}}$ vs log $D_{\mathrm{E,COase}}$ values. The scale bars in yellow measure 5 μm in images (a)-(c), (e) and (f). This figure uses cell 1 in Table 2. The reported values are averages obtained from analysis of an entire cell. The analyses were performed on 3 cells from 3 different batches of cells with similar results (Table 2).

14 Appendix

14.1 Plasmids utilized in this study

The plasmids used in this study are shown here.

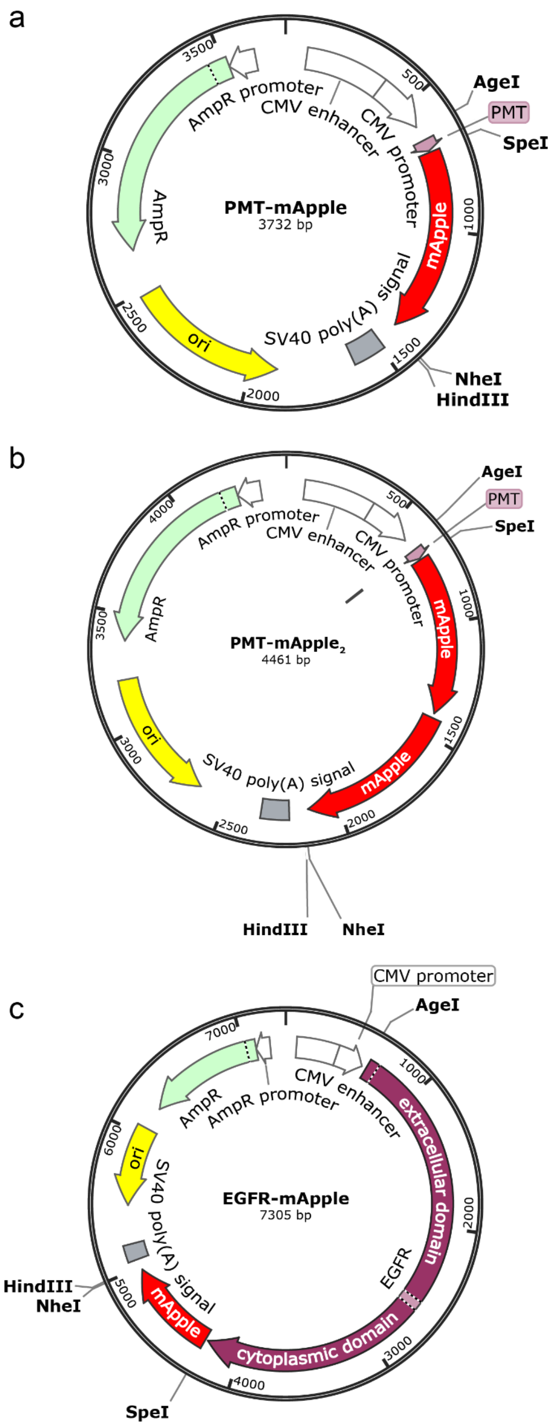

Supplementary Fig. 21: Plasmid maps of calibration probes and EGFR-mApple: Plasmid maps of PMT-mApple, PMT-mApple$_2$, and EGFR-mApple are shown in images **(a)**, **(b),** and **(c)**, respectively. Some of the major sequences are labelled, along with 4 restriction sites – AgeI, SpeI, NheI, and HindIII.

14.2 Brightness of nth order oligomer species

The simplification of the formula derived for the brightness of an oligomer of order *n* (section 3.4) is shown here.

$$B_n = \frac{N_n \varepsilon^2 \sum_{i=1}^{n} i^2 \binom{n}{i} p^i (1-p)^{n-i}}{N_n \varepsilon \sum_{i=1}^{n} i \binom{n}{i} p^i (1-p)^{n-i}} = \frac{N_n \varepsilon^2 np\big((n-1)p+1\big)}{N_n \varepsilon np} = \varepsilon\big((n-1)p+1\big)$$

$$(y+x)^n = \sum_{i=0}^{n} \binom{n}{i} x^{n-i} y^i$$

$$\frac{\partial}{\partial y}(y+x)^n = \sum_{i=1}^{n} i \binom{n}{i} x^{n-i} y^{i-1}$$

$$ny(y+x)^{n-1} = \sum_{i=1}^{n} i \binom{n}{i} x^{n-i} y^i$$

Substituting, $x = 1\text{-}p$ and $y = p$, the denominator simplifies to np.

$$\frac{\partial}{\partial y} ny(y+x)^{n-1} = \sum_{i=1}^{n} i^2 \binom{n}{i} x^{n-i} y^{i-1}$$

$$ny^2(n-1)(y+x)^{n-2} + ny(y+x)^{n-1} = \sum_{i=1}^{n} i^2 \binom{n}{i} x^{n-i} y^i$$

Substituting, $x = 1\text{-}p$ and $y = p$, the numerator simplifies to $np^2(n-1) + np = np\big((n-1)p+1\big)$.

15 Supplementary References

1 Gustafsson, N. *et al.* Fast live-cell conventional fluorophore nanoscopy with ImageJ through super-resolution radial fluctuations. *Nature Communications* **7**, 12471, doi:10.1038/ncomms12471 (2016).

2 Dertinger, T., Colyer, R., Iyer, G., Weiss, S. & Enderlein, J. Fast, background-free, 3D super-resolution optical fluctuation imaging (SOFI). *Proceedings of the National Academy of Sciences* **106**, 22287-22292, doi:10.1073/pnas.0907866106 (2009).

3 Kannan, B. *et al.* Spatially resolved total internal reflection fluorescence correlation microscopy using an electron multiplying charge-coupled device camera. *Anal. Chem.* **79**, 4463-4470, doi:10.1021/ac0624546 (2007).

4 Wohland, T., Shi, X., Sankaran, J. & Stelzer, E. H. K. Single Plane Illumination Fluorescence Correlation Spectroscopy (SPIM-FCS) probes inhomogeneous three-dimensional environments. *Opt. Express* **18**, 10627-10641 (2010).

5 Wawrezinieck, L., Rigneault, H., Marguet, D. & Lenne, P. F. Fluorescence correlation spectroscopy diffusion laws to probe the submicron cell membrane organization. *Biophys J* **89**, 4029-4042, doi:10.1529/biophysj.105.067959 (2005).

6 Veerapathiran, S. & Wohland, T. The imaging FCS diffusion law in the presence of multiple diffusive modes. *Methods* **140-141**, 140-150, doi:https://doi.org/10.1016/j.ymeth.2017.11.016 (2018).

7 Digman, M. A., Dalal, R., Horwitz, A. F. & Gratton, E. Mapping the number of molecules and brightness in the laser scanning microscope. *Biophys J* **94**, 2320-2332, doi:10.1529/biophysj.107.114645 (2008).

8 Unruh, J. R. & Gratton, E. Analysis of molecular concentration and brightness from fluorescence fluctuation data with an electron multiplied CCD camera. *Biophysical journal* **95**, 5385-5398, doi:10.1529/biophysj.108.130310 (2008).

9 Foo, Y. H., Naredi-Rainer, N., Lamb, D. C., Ahmed, S. & Wohland, T. Factors affecting the quantification of biomolecular interactions by fluorescence cross-correlation spectroscopy. *Biophysical journal* **102**, 1174-1183, doi:10.1016/j.bpj.2012.01.040 (2012).

10 Dunsing, V. *et al.* Optimal fluorescent protein tags for quantifying protein oligomerization in living cells. *Scientific Reports* **8**, 10634, doi:10.1038/s41598-018-28858-0 (2018).

11 Cranfill, P. J. *et al.* Quantitative assessment of fluorescent proteins. *Nature Methods* **13**, 557-562, doi:10.1038/nmeth.3891 (2016).

12 Przybylski, A., Thiel, B., Keller-Findeisen, J., Stock, B. & Bates, M. Gpufit: An open-source toolkit for GPU-accelerated curve fitting. *Scientific Reports* **7**, 15722, doi:10.1038/s41598-017-15313-9 (2017).

13 Koho, S. *et al.* Fourier ring correlation simplifies image restoration in fluorescence microscopy. *Nature Communications* **10**, 3103, doi:10.1038/s41467-019-11024-z (2019).

14 Banterle, N., Bui, K. H., Lemke, E. A. & Beck, M. Fourier ring correlation as a resolution criterion for super-resolution microscopy. *Journal of Structural Biology* **183**, 363-367, doi:https://doi.org/10.1016/j.jsb.2013.05.004 (2013).

15 Hirsch, M., Wareham, R. J., Martin-Fernandez, M. L., Hobson, M. P. & Rolfe, D. J. A Stochastic Model for Electron Multiplication Charge-Coupled Devices – From Theory to Practice. *PLOS ONE* **8**, e53671, doi:10.1371/journal.pone.0053671 (2013).
